# Latitudinal gradient of cyanobacterial diversity in tidal flats

**DOI:** 10.1371/journal.pone.0224444

**Published:** 2019-11-13

**Authors:** Janina C. Vogt, Raeid M. M. Abed, Dirk C. Albach, Katarzyna A. Palinska

**Affiliations:** 1 Institute for Biology and Environmental Science (IBU), Biodiversity and Evolution of Plants, Carl-von-Ossietzky University of Oldenburg, Oldenburg, Germany; 2 Biology Department, College of Science, Sultan Qaboos University, Al Khoud, Muscat, Sultanate of Oman; 3 Department of Marine Biology and Ecology, Institute of Oceanography, University of Gdansk, al. Marszałka Józefa Piłsudskiego 46, Gdynia, Poland; INRA, FRANCE

## Abstract

Latitudinal diversity gradients are well-known for plants and animals, but only recently similar patterns have been described for some specific microbial communities in distinct habitats. Although microbial diversity is well-investigated worldwide, most of the studies are spatially too restricted to allow general statements about global diversity patterns. Additionally, methodological differences make it hard and often impossible to compare several studies. This study investigated the cyanobacterial diversity in tidal flats along geographical and ecological gradients based on high-throughput sequencing of 16S rRNA gene fragments (Illumina MiSeq) and environmental data on a large spatial scale from the subtropics to the Arctic. Latitude and strongly correlated environmental parameters (e.g. temperature) were identified as important drivers of cyanobacterial diversity on global scale resulting in a latitudinal diversity gradient similar to that known from plants and animals. Other non-correlated parameters (e.g. grain size) were shown to be more important on local scales, although no consistent pattern occurred across different locations. Among a total number of 989 operational taxonomic units (OTUs) only one cosmopolitan (classified as *Coleofasciculus chthonoplastes*), but many location-specific and putative endemic ones (78%) were detected. High proportions of rare members of the community (up to 86%) were found in all samples. Phylogenetic beta diversity was shown to be influenced by the developmental stage of the mat community becoming increasingly similar with increasing stabilization.

## Introduction

Detailed analyses of microbial diversity have become common during the last years based on cost-efficient high-throughput sequencing (HTS) technologies, which allow deep insights into the community structure of environmental samples including the previously undetected ‘rare biosphere’. Rare microbial species have been shown to be extremely diverse and significantly contributed to the increase in richness and diversity of communities [1]. Whereas studies focusing on local communities increased our knowledge of the microbial diversity worldwide [2; 3], biogeographical patterns only start to emerge with an increasing use of HTS technologies.

In the beginning of the 20^th^ century, Beijerinck [4] and Baas-Becking [5] established the hypothesis that ‘*Everything is everywhere*, *but the environment selects*’, which is commonly assumed for free-living microorganisms. Thus, microorganisms are supposed to be ubiquitous and disperse freely without geographical barriers or isolation as supported by studies of benthic and planktonic protists [6; 7; 8; 9]. Within the phylum Cyanobacteria such cosmopolitan distribution patterns were shown for some species e.g. *Coleofasciculus* (*Microcoleus*) *chthonoplastes* [10], *Mastigocladus laminosus* [11; 12], *Cylindrospermopsis raciborskii* [13; 14; 15], and *Microcystis aeruginosa* [16; 17]. Recent high-resolution studies of microbial diversity found evidence for unique biogeographical distribution patterns of microorganisms on different spatial scales [18; 19; 20; 21] and several molecular studies observed them for specific cyanobacterial taxa [22] e.g. in hot springs [23] and ice-covered lakes in the Antarctica [24].

Although biogeographical patterns and the related gradients in biodiversity have been well investigated in plants and animals for centuries [25; 26; 27; 28; 29; 30; 31], there is still less known about microbial and especially prokaryotic biogeography [22; 32; 33; 34; 35].

One of the oldest and most commonly observed patterns of worldwide biodiversity is the latitudinal gradient, which is characterized by decreasing species richness from the tropics to the poles. Latitudinal gradients have been demonstrated to exist in diverse higher eukaryotic taxa [36; 37; 38; 39; 40; 41; 42; 43]. Latitude and correlated parameters, such as climate (temperature), are commonly supposed to determine biodiversity for historical (age/area), evolutionary (speciation/diversification rates), and ecological reasons (ecological/physiological opportunities, distribution ranges) [43; 44]. Based on that the tropics are commonly considered to be a “cradle and museum of biodiversity” that spread out more or less successfully towards the poles [39; 44]. However, recent studies showed that high ecological opportunity and high speciation rates are not necessarily restricted to the tropics [45; 46] with one study having found evidence for habitat-specific variations within this general latitudinal diversity gradient [47].

Due to the immense population size, a tremendous dispersal potential, and diverse physiological properties of microorganisms, not all mechanisms shaping biogeography of macroorganisms, such as speciation, extinction, dispersal, and longevity, are directly adaptable to microorganisms, even if they are not fundamentally different. Environmental gradients and habitat types are supposed to become much more important for their geographical distribution than distances [32; 34; 48].

Recent studies verified latitudinal diversity gradients for terrestrial and marine/planktonic bacteria [49; 50; 51; 52] and suggested biogeographical patterns for prokaryotes similar to those described for eukaryotes according to their physiological properties [11; 32; 52]. However, most studies on microbial diversity are spatially too restricted to allow general statements about global patterns [24; 53; 54].

Tidal flats occur worldwide along low-sloped coastlines with non-vegetated mud/sand plains in sheltered/low-energy marine environments (bays, lagoons, estuaries, back-barrier of islands), or open coasts [55; 56; 57]. Strongly fluctuating environmental conditions due to tidal activity with periodic inundation and desiccation are characteristics of these highly dynamic coastal ecosystems at the transition from terrestrial to marine environments. Tidal flats provide extreme, but comparable environmental conditions at geographically distant sites differing mainly in climate conditions. Microbial communities in tidal flats usually form thin biofilms or more or less stabilized, laminated microbial mats, where microorganisms are arranged according to internal physicochemical gradients [58; 59; 60]. Cyanobacteria are important members of these benthic microbial mats as pioneer organisms, main primary producers, and stabilizers [58; 59; 60; 61]. Diverse environmental parameters influence the successive formation of such mat communities and their composition, e.g. temperature, grain size, salinity, and nutrient composition [58; 62; 63]. Since tidal flats are spatially separated without strict boundaries and inhabited by a highly diverse microbial community [61; 64; 65], they are a promising habitat for an investigation of biodiversity patterns of cyanobacterial populations.

Based on the ecological importance and variability of this habitat as well as its worldwide distribution, we investigated cyanobacterial diversity patterns along geographical and ecological gradients in different climate zones ((sub)arctic to subtropical-arid) within a defined and comparable habitat. We compared diversity measures based on high-throughput sequencing of 16S rRNA gene fragments from tidal flats at five geographically distant sites (Iceland, France, Germany, Croatia, and Oman), including samples that differed in grain size, salinity, alkalinity, and nutrient composition. This data allowed us to test the two most commonly inferred hypotheses about microbial biogeography: the “Everything is everywhere”-hypothesis and the latitudinal biodiversity gradient hypothesis, with unprecedented precision for such organisms based on comparable sampling and sequencing methodologies.

Specifically, we examined the following hypotheses: 1) cyanobacterial species from tidal flats are cosmopolitan across latitude rather than forming climate zone-specific communities; 2) the diversity of cyanobacterial communities decrease with latitude similar to that of plants and animals; 3) environmental parameters such as grain size, salinity, total alkalinity and nutrients show similar effects on community diversity across different climate zones.

## Materials and methods

### Study sites and sampling

**Fig 1:**
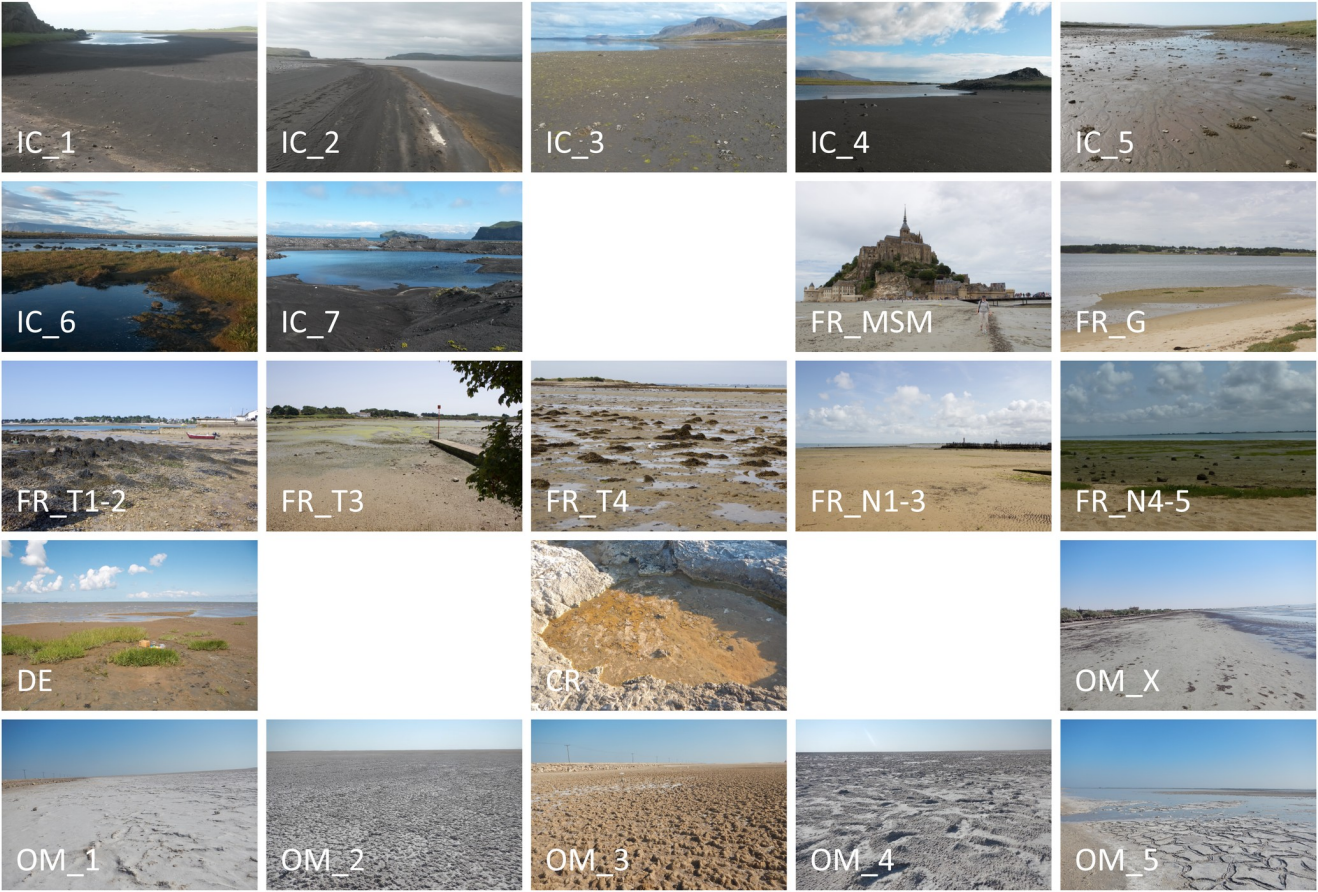
Pictures of all sampling sites. CR = Croatia, FR = France, DE = Germany, IC = Iceland, OM = Oman.

Study sites were located in tidal flats in different climate zones: (sub)arctic (Iceland), temperate (Germany, France (Brittany)), subtropical-dry-summer (Croatia), and subtropical-arid (Oman) (Fig 1, S1 Table). The necessary permissions to access the study sites and collect samples there were acquired from the Nationalparkverwaltung “Niedersächsisches Wattenmeer” for the study sites in Germany and from Sultan Qaboos University within the framework of a joint scientific collaboration project for the study sites in Oman. For study sites in Iceland, France, and Croatia, the national focal points were contacted and no specific permits were required. The field studies did not involve any endangered or protected species. As previously described by Vogt *et al*. [62; 63], the upper part of the sediment (ca. 1 cm) including the entire phototrophic part of the microbial community was sampled at low tide using sterile Petri dishes (2–3 replicates per sample within an area of about 1m^2^). Pore water samples were collected directly at the same positions using Rhizons (Rhizosphere Research Products, Wageningen, NL) that were placed horizontally in the upper 5 mm of the sediment and extracted pore water through a filter membrane with the help of vacuum syringes. Open water samples were collected at incoming tide with 500 ml plastic bottles and were sterile filtered afterwards. All sediment and water samples were taken once at each study site between summer 2013 and summer 2015 (S1 Table).

### Analysis of abiotic parameters

Open and pore water salinity was measured directly in the field using a hand-held refractometer (arcarda Handelsgesellschaft, Reichelsheim, Germany) and pH of the open water samples as soon as possible in the lab with a pH/mV hand-held meter pH330 (WTW, Weilheim, Germany). Total alkalinity (modified according [66]) and nutrient concentrations were measured spectrophotometrically (partially modified and adapted after: [67] (NH_4_); [68; 69] (NO_2_, NO_x_); [70; 71; 72] (PO_4_)) using a Fluostar Optima Reader (BMG LABTECH GmbH, Ortenberg, Germany).

For grain size measurements, the sediment samples were dried in a compartment drier at 40°C for at least 8 h, ground with mortar and pestle and sieved through a 2 mm screen. Particles larger than 2mm in diameter were sorted out and disregarded for further measurements. Sieved sediment was pre-treated with 10% (v/v) HCl to remove carbonates and 30% H_2_O_2_ to remove organic matter. Grain size was measured with a laser particle sizer Analysette 22 MicroTec plus with Wet Disperser Unit navigated by the MaS Control Software (Fritsch, Idar-Oberstein, Germany) in three replicates per sample and twice per replicate. The mean grain size of 90% of all particles was used to classify four grain size fractions according to the DIN EN ISO 14688–1 norm: CSa = coarse sand (0.63–2.0mm), MSa = medium sand (0.2–0.63mm), FSa = fine sand (0.063–0.2mm), CSi = coarse silt (0.02–0.063mm).

The stabilization level of all samples was qualitatively classified into three classes: high (well-stabilized and laminated microbial mats that can be peeled off the sediment in large pieces (> 25 cm^2^)), medium (slightly stabilized sediment without clear laminations, that cannot be peeled off the sediment in large, but smaller pieces), and low (lose sediment without notably amounts of stabilizing compounds).

Climate data (air temperature at 10 m above the surface of the earth (°C), precipitation (mm/day) as averaged monthly values over a 22-years period (Jul 1983—Jun 2005)) was obtained from the Atmospheric Science Data Center/NASA Langley Research Center (https://eosweb.larc.nasa.gov/sse/, accessed 09/2017), seawater temperatures from www.seatemperature.org (accessed 08/2017). Annual mean values were calculated based on the monthly averaged values (Table 1).

**Table 1:** Climatic characteristics of the sampled locations.

Location	sampling season	climate zone	months >5°C	months > 10°C	annual mean temperature [°C]	annual mean water temperature [°C]	annual mean precipitation [mm/day]
Iceland	summer	(sub)arctic	5	1	3.9	8.0	4.1
Germany	summer	temperate	8	6	9.6	11.2	2.9
France	summer	temperate	12	7	13.0	14.1	2.2
Croatia	summer	subtropical/ dry summer	9	7	13.4	18.6	2.8
Oman	winter	subtropical/ arid	12	12	27.7	27.3	0.3

Number of months with more than 5°C/10°C represent the length of the respective vegetation periods. Climate data: https://eosweb.larc.nasa.gov/sse/ (averaged monthly values from 1983–2005), accessed 21.09.2017; water temperatures (estimated from values of nearby locations): www.seatemperature.org, accessed 02.08.2017. All values represent the average of the sampling sites per location.

Linear distances between sampling sites were calculated based on the measured coordinates using google maps (https://www.google.com/maps).

### Molecular work and sequence analysis

Total DNA of sediment samples (pooled triplicates) was isolated using the UltraClean Microbial DNA Isolation Kit (MO BIO Laboratories, Inc., Carlsbad, CA, USA) for all samples except the ones collected in Oman. It was not possible to reach sufficient DNA concentrations for the Oman samples due to their hypersalinity using the UltraClean Microbial DNA Isolation Kit, despite additional dilution steps. Thus, we had to switch to the Power Biofilm DNA Isolation Kit (MO BIO Laboratories, Inc., Carlsbad, CA, USA), which used alternative buffers, but the same filter columns. In both cases, the manufacturers protocol was extended by 10 freeze and thaw cycles (liquid nitrogen/60°C water bath) and an overnight incubation with Proteinase K during the lysis process. Pre-selective PCR was performed with cyanobacteria-specific CYA106F [73] and universal bacterial 16S1494R [74] primers and the REDTaq Ready Mix (Sigma-Aldrich Chemie GmbH, Munich, Germany)). PCR products were purified using the QIAquick PCR Purification Kit (QIAgen, Hilden, Germany). Details of the entire DNA preparation process were previously described by Vogt *et al*. [62; 63].

Sequencing of 16S rRNA gene fragments with cyanobacteria specific primers (CYA359F/CYA781R [73]) was conducted in two runs (samples from Oman and all other samples) at the Research and Testing Laboratory (Lubbock, Texas, USA) using the Illumina MiSeq technology.

All sequences were checked for quality by trimming off primer sequences, removing all sequences with mismatches in primer sequences, ambiguous bases, homopolymers longer than 8 bp, and/or average quality scores below 25 in a 50 bp sliding window and screening for a defined fragment length (250–450 bp) using MOTHUR v. 1.36.1 [75] as described by Vogt *et al*. [62; 63]. The remaining quality checked sequences were aligned against the SILVA reference dataset (Release 102, http://www.arb-silva.de/, [76]), modified for bacterial sequences (silva.bacteria reference alignment, http://www.mothur.org/wiki/Silva_reference_files (accessed 04/2012)) and combined with an additional cyanobacterial rRNA dataset from Herdman (http://cyanophylo.blogspot.de/, downloaded June 2014). Identical sequences were condensed and sequences with up to 4 bp difference to each other were additionally pre-clustered (MOTHUR v.1.36.1 [75]). Chimeric sequences were detected using the VSEARCH command (de novo, dereplicated) based on the UCHIME algorithm [77; 78] and removed (MOTHUR v.1.39.1 [75]). The VSEARCH distance-based greedy clustering method (de novo) was used to create operational taxonomic units (OTUs) at a 97% similarity level (MOTHUR v.1.39.1 [75; 78; 79]). OTUs with less than 11 sequences within the entire dataset were excluded. Representative consensus sequences of each OTU (> 10 sequences) were classified using NCBI Nucleotide Blast and the ‘16S ribosomal RNA sequences (Bacteria and Archaea)’ database (https://blast.ncbi.nlm.nih.gov, [80]). The taxonomic information of the best match (highest identity, max/total score, query cover) was adopted in a resolution depending on the identity values: 85%, 90% and 95% identity for phyla, orders, and genera, respectively. Only cyanobacterial sequences (at least 85% similarity) were analyzed further. Representative sequences of all cyanobacterial OTUs were deposited in the NCBI GenBank database (https://www.ncbi.nlm.nih.gov/Genbank/) with accession numbers MF507041-MF508244.

Relative sequence abundances were calculated based on the total number of sequences and OTUs per sample. OTUs that included less than 0.1% of all quality checked sequences per sample were considered as rare OTUs.

### Alpha and beta diversity analysis

OTU-based alpha and beta diversity estimates were calculated in MOTHUR v.1.39.1 [75] based on all quality checked sequences per sample and described as richness (observed (sobs) and chao1 (chao) estimated numbers of OTUs) and diversity (Shannon diversity index (shannon)), as well as shared richness (observed (sharedsobs) numbers of shared OTUs) and dissimilarity coefficients (Bray-Curtis (braycurtis) and Jaccard (jclass) dissimilarity coefficients). The shared observed richness values per sample (sharedsobs calculated in MOTHUR v.1.39.1 [75]) were summarized per location as presence/absence matrix and a VENN diagram was constructed (manually) based on these summarized values. Rarefaction curve data for observed OTUs per sample were calculated in MOTHUR v.1.39.1 [75] with 1000 randomizations using all available quality checked sequences and a re-sampling without replacement approach.

A maximum likelihood tree with rapid bootstraps (100 replicates, GTRGAMMA) was constructed in RAxML Version 8 [81] (raxmlGUI version 1.5b1 [82]) for the representative sequences of all 989 OTUs. Based on this tree the phylogenetic diversity was analyzed in R version 3.3.1 [83] using the ‘picante’ package (version 1.6–2) [84]. Faith’s phylogenetic diversity (PD [85] = total of the unique branch length in the tree), mean pairwise distance (MPD [86], sequence abundance weighted), and MPD between pairs of communities (phylogenetic beta diversity) were calculated according to the developer’s instructions (http://picante.r-forge.r-project.org/picante-intro.pdf (accessed 04/2010)).

To identify potential linear correlations between different environmental/geographical parameters and diversity values, regression analyses were performed in R version 3.3.1 using the linear model function for linear regression [83].

Redundancy analyses (RDA) based on sequence abundances per OTU and standardized environmental parameters were performed in R version 3.6.0 [87] using the ‘vegan’ package (version 2.5–5) [88]. Sequence numbers per sample and OTU (+ 0.5 to avoid zero values) were centered log-ratio (clr) transformed using the ‘compositions' package (version 1.40–2) [89]. Non- and less-correlated environmental parameters were selected according to their collinearity (pairwise correlation (Pearson) < 0.8 or > - 0.8). Dependent parameters (e.g. NO_2_ + NO_3_ = NO_X_) were removed. Variance inflation factors of the selected parameters were checked to be below 10. Forward model selection using ordiR2step method of the R package ‘vegan’ [88] was performed to identify the best environmental predictors for OTU distribution/occurrence. Total and pure effects of the environmental parameters were calculated using permuRDAv1.6.R (https://github.com/chassenr/ARISA) to quantify the significance of selected environmental parameters explaining the variation in cyanobacterial community composition.

Principal Coordinates Analyses (PCoAs) based on Jaccard and Bray-Curtis dissimilarity coefficients (OTU-based) as well as the phylogenetic beta diversity were calculated in MOTHUR v.1.39.1 [75] to illustrate the dissimilarity of the sampling sites and identify potential distribution patterns based on different diversity estimates considering the presence (Jaccard), abundance (Bray-Curtis), and phylogenetic relationship (phylogenetic beta diversity) of the detected OTUs.

All statistical tests (Shapiro-Wilk normality test, Kruskal-Wallis test, ANOVA) were performed in R version 3.3.1 [83] or version 3.6.0 [87]. P-values < 0.05 were considered as significant.

## Results

### Cyanobacterial richness and diversity

A total number of 425,730 sequences from 24 samples were clustered into 989 cyanobacterial OTUs (> 10 sequences, 97% similarity). OTU- and phylogeny-based richness (sobs, Chao1, PD) and diversity (Shannon, MPD) estimates per sample, as well as the relative abundance of rare OTUs (< 0.1% of all sequences per sample) varied strongly among all samples (Table 2, S1 Fig). All richness and diversity estimates were calculated based on the complete dataset, including all available quality-checked sequences per sample without normalization that would have removed 90% of the final sequences and 10% of the observed OTUs. Detailed sequence counts during sequence analysis including a putative normalization (S2 Table) and rarefaction curves of all samples (S2 Fig) are shown as Supplementary Information.

**Table 2:** OTU- and phylogeny-based alpha diversity.

location	group	nseqs	sobs	rel. abund. rare OTUs	Chao1	Shannon	PD	MPD
**Iceland**	**IC_1**	5823	16	0.44	19	0.6	3.7	0.29
	**IC_2**	5053	25	0.32	26	1.1	4.1	0.14
	**IC_4**	1863	37	0.16	40	1.5	6.6	0.43
	**IC_5**	15570	108	0.55	115	2.1	10.1	0.33
	**IC_6**	6432	48	0.44	59	0.8	6.6	0.09
	**IC_3**	3607	20	0.30	22	1.4	4.1	0.62
	**IC_7**	9131	23	0.13	26	1.5	3.9	0.25
**Germany**	**DE_cg**	12592	113	0.68	125	1.5	11.1	0.25
	**DE_sa**	6962	55	0.45	64	1.3	4.4	0.33
	**DE_si**	8087	73	0.63	75	0.9	7.9	0.10
**France**	**FR_T4**	6883	124	0.39	183	3.1	12.6	0.75
	**FR_G**	2228	56	0.30	62	1.9	6.1	0.33
	**FR_N1_3**	3558	60	0.47	82	1.7	6.6	0.33
	**FR_T1_2**	9678	76	0.36	94	1.9	10.8	0.39
	**FR_MSM**	7502	36	0.11	37	1.9	6.2	0.33
	**FR_N4_5**	11300	63	0.43	69	1.4	6.1	0.11
	**FR_T3**	14648	90	0.70	97	1.4	9.5	0.18
**Croatia**	**CR**	22063	53	0.51	56	0.8	4.3	0.05
**Oman**	**OM_X**	71549	174	0.76	179	2.0	14.1	0.33
	**OM_1**	60953	154	0.86	175	1.4	11.5	0.11
	**OM_3**	23074	204	0.74	225	2.5	14.3	0.27
	**OM_2**	12647	276	0.68	323	3.1	18.9	0.42
	**OM_4**	57341	179	0.82	198	2.0	11.5	0.22
	**OM_5**	47186	135	0.77	149	1.8	10.2	0.16
**pvalue**	**Kruskal-Wallis**_**loc**_	**0.007**	**0.002**	**0.004**	**0.003**	**0.040**	**0.007**	**0.348**

OTU-based alpha diversity described as observed (sobs) and Chao1 estimated richness and Shannon diversity index and phylogeny-based alpha diversity described as Faith’s phylogenetic diversity (PD) and the abundance weighted mean pairwise distance (MPD) based on the maximum likelihood tree of representative sequences per OTU; rare OTUs include less than 0.1% of the sequences per sample; nseqs = number of sequences; CR = Croatia, FR = France, DE = Germany, IC = Iceland, OM = Oman. Kruskal-Wallis tests were performed to identify significant differences of the parameters between locations.

**Fig 2:**
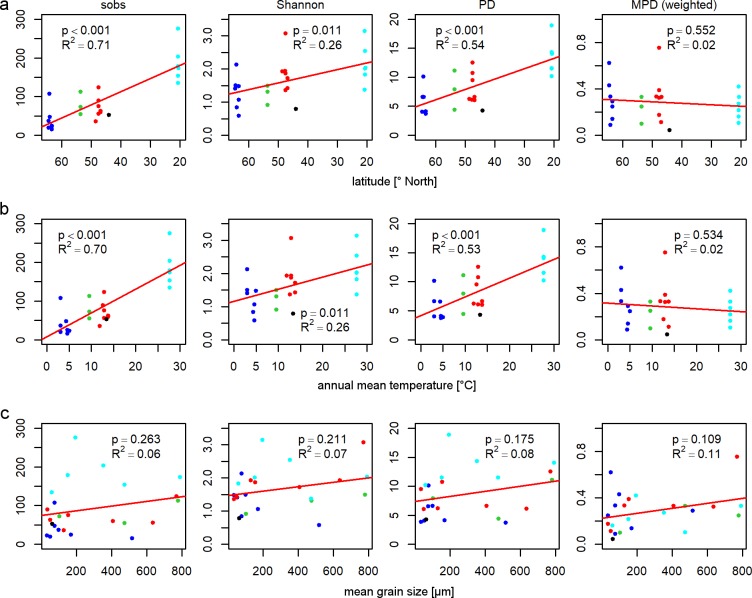
**Linear regression analyses of OTU- and phylogeny-based alpha diversity estimates in relation to latitude (a), annual mean temperature (b), and mean grain size (c).** OTU-based alpha diversity was described as observed richness (sobs) and Shannon diversity (Shannon), phylogeny based alpha diversity as Faith’s phylogenetic diversity (PD), and Mean pairwise distances (MPD, abundance weighted) for representative sequences per OTU based on a maximum likelihood tree; annual mean temperatures adopted from https://eosweb.larc.nasa.gov/sse/ (accessed 21.09.2017) as averaged monthly values of a 22-years period (1983–2005); colors code locations: dark blue = Iceland, green = Germany, red = France, black = Croatia, light blue = Oman.

A latitudinal diversity gradient with significant linear correlations (negative) of richness (OTU- and phylogeny-based, p < 0.001) and OTU-based diversity values (Shannon index, p < 0.05) was verified here (Fig 2A). However, phylogenetic diversity (MPD) showed no significant correlation to latitude. As expected, the latitudinal gradient of sampling sites was strongly correlated (p < 0.001) to climate-related parameters (i.e. annual mean temperature, precipitation, water temperature, and the length of the vegetation period (> 10°C) (S3 Fig)). Therefore, similar trends in alpha diversity were observed for these climate-related parameters as shown here for annual mean temperatures with increasing richness (sobs, PD, p < 0.001) and OTU-based diversity (Shannon index, p < 0.05) with increasing temperature (Fig 2B, positive correlations) and decreasing precipitation (S5 Table, negative correlation). No significant correlations to temperature were observed for phylogenetic diversity (MPD). Only a minimal trend of increasing alpha diversity (including the phylogenetic diversity (MPD)) with increasing grain size could be detected (Fig 2C).

Apart from different climate conditions, the results demonstrated that tidal flats differ clearly in environmental parameters such as salinity (from 0.2 to 18%), grain size (from 33.2 to 790.7 μm), TA (from 0.4 to 2.9 mM), and sediment stabilization (Table 3). Detailed results of nutrient measurements in pore and seawater samples are available as supplementary data (S3 Table). Significant linear correlations (p < 0.05) between different environmental parameters were detected by regression analyses e.g. for latitude and climatic parameters, as well as for seawater salinity, TA, NH_4_ NO_3_, NO_X_, and pore water salinity (S4 Table). Expected correlations of nutrient availability and grain size [58; 90] could not be verified here (S4 Table). As shown by linear regression, analyses richness values (sobs, PD) were best correlated to latitude, temperature, precipitation, salinity, and alkalinity (p < 0.001), while the correlations of diversity estimates (Shannon, MPD) to all measured abiotic parameters were much weaker or were nearly absent (0.01 < p < 1) (Fig 2, S5 Table).

**Table 3:** Sediment and water characteristics of the sampling sites.

location	sample	grain size [μm]	sediment type	level of stabilization	Salinity [%]	TA [mM]
**Iceland**	**IC_1**	516.5	MSa	medium	0.2	0.4
	**IC_2**	168.7	FSa	low	3.1	1.8
	**IC_4**	98.8	FSa	low	3.2	1.8
	**IC_5**	76.3	FSa	low	2.5	1.4
	**IC_6**	75.0	FSa	high	3.9	1.9
	**IC_3**	51.0	CSi	low	0.9	0.3
	**IC_7**	33.2	CSi	medium	3.1	2.2
**Germany**	**DE_cg**	778.7	CSa	medium	3.5	2.9
	**DE_sa**	474.2	MSa	high	3.5	2.9
	**DE_si**	101.9	FSa	medium	3.5	2.9
**France**	**FR_T4**	769.9	CSa	low	3.7	2.1
	**FR_G**	634.6	CSa	low	3.7	2.1
	**FR_N1-3**	406.9	MSa	medium	3.7	2.1
	**FR_T1-2**	153.6	FSa	medium	3.6	2.1
	**FR_MSM**	128.4	FSa	low	3.7	1.1
	**FR_N4-5**	49.3	CSi	low	3.8	2.2
	**FR_T3**	33.5	CSi	high	3.7	2.1
**Croatia**	**CR**	62.1	CSi	high	3.9	NA
**Oman**	**OM_X**	790.7	CSa	low	5.4	3.0
	**OM_1**	473.0	MSa	low	18.0	3.6
	**OM_3**	352.3	MSa	medium	18.0	3.6
	**OM_2**	193.9	FSa	medium	18.0	3.6
	**OM_4**	151.2	FSa	high	18.0	3.6
	**OM_5**	58.7	CSi	high	18.0	3.6
**p-values**	**Kruskal-Wallis**	**0.318 (loc)** **< 0.001 (sed)**			**< 0.001 (loc)** **0.979 (sed)**	**< 0.001 (loc)** **0.752 (sed)**

Mean grain size of 90% of all particles classified in grain size fractions (sediment types) according to DIN EN ISO 14688–1: CSa = coarse sand (0.63–2.0mm), MSa = medium sand (0.2–0.63mm), FSa = fine sand (0.063–0.2mm), CSi = coarse silt (0.02–0.063mm); stabilization level of sediment samples qualitatively classified as low = loose sediment covered by thin biofilms, medium = sediment surface stabilized by EPS, high = well stabilized and laminated microbial mats; salinity and total alkalinity (TA) in sea water samples; CR = Croatia, FR = France, DE = Germany, IC = Iceland, OM = Oman. Kruskal-Wallis tests were performed to identify significant differences of the parameters between locations (loc) or sediment types (sed).

### Community composition and distribution

**Fig 3:**
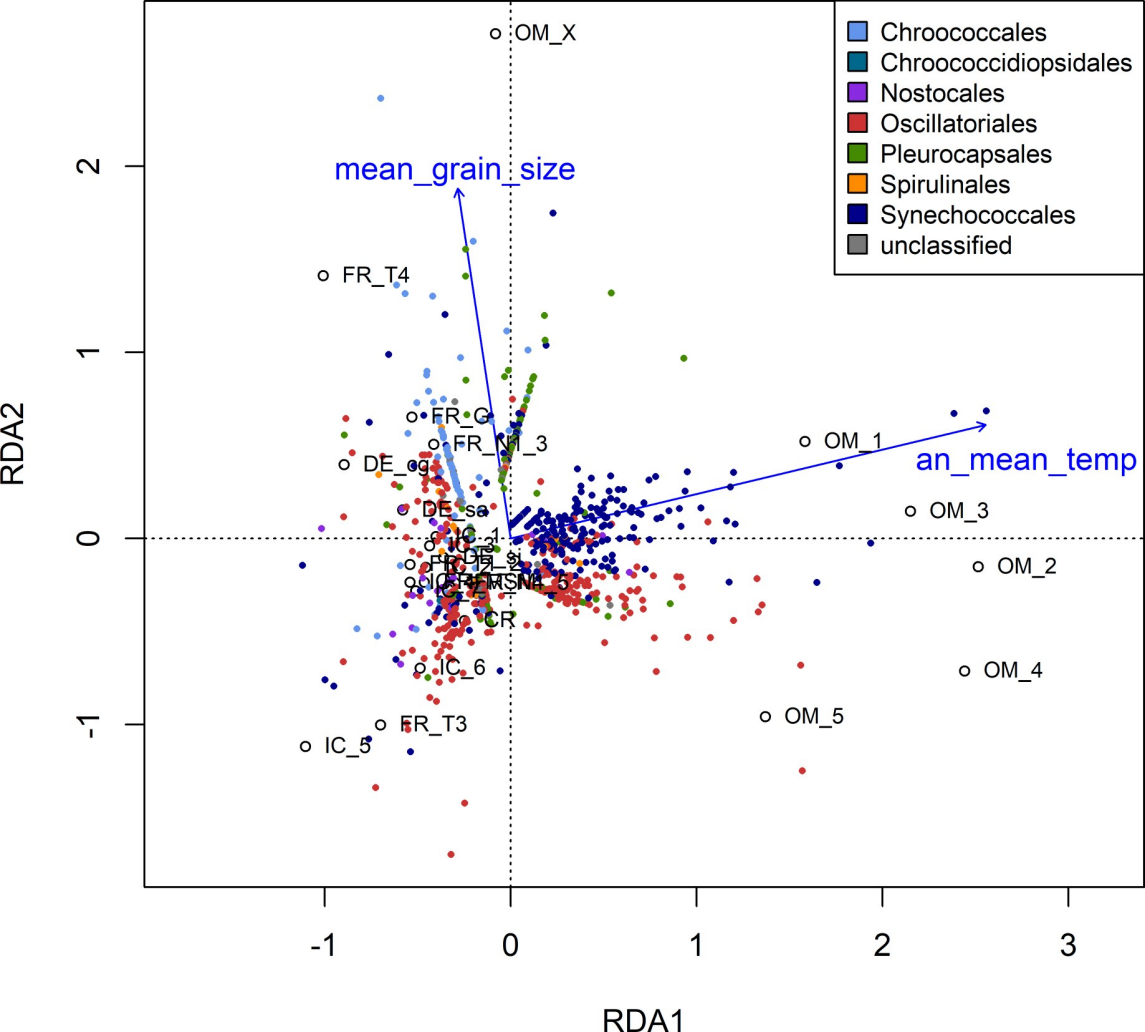
RDA analysis with forward model selection based on sequence numbers per OTU and a subset of non- or less-correlated abiotic parameters. Standardized values of annual mean temperature, mean grain size, pore water NH_4_/NO_X_/PO_4_; IC = Iceland, DE = Germany, FR = France, CR = Croatia, OM = Oman; RDA1 axis explains 13%, RDA2 axis 7% of the variance. Total and pure effects of the environmental parameters are provided as Supplementary Information (S6 Table).

The influence of environmental parameters on the distribution of OTUs (sequence numbers per OTU and sample, centered log-ratio transformed) was shown via RDA analysis (Fig 3) based on a subset of non- or less-correlated abiotic parameters (standardized values of annual mean temperature, mean grain size, and pore water NH_4_/NO_X_/PO_4_). The forward model selection revealed the annual mean temperature and mean grain size as the most important factors influencing cyanobacterial OTUs distribution. Slight taxon-specific patterns could be observed. Whereas Oscillatoriales OTUs were scattered along both RDA axes, the predominantly unicellular Chroococcales and Pleurocapsales OTUs were enriched in samples with larger grain size, while Synechococcales OTUs were abundant in samples with higher temperature. No clear patterns were distinguishable for OTUs classified as Chroococcidiopsidales, Nostocales, Spirulinales, and unclassified cyanobacteria.

**Fig 4:**
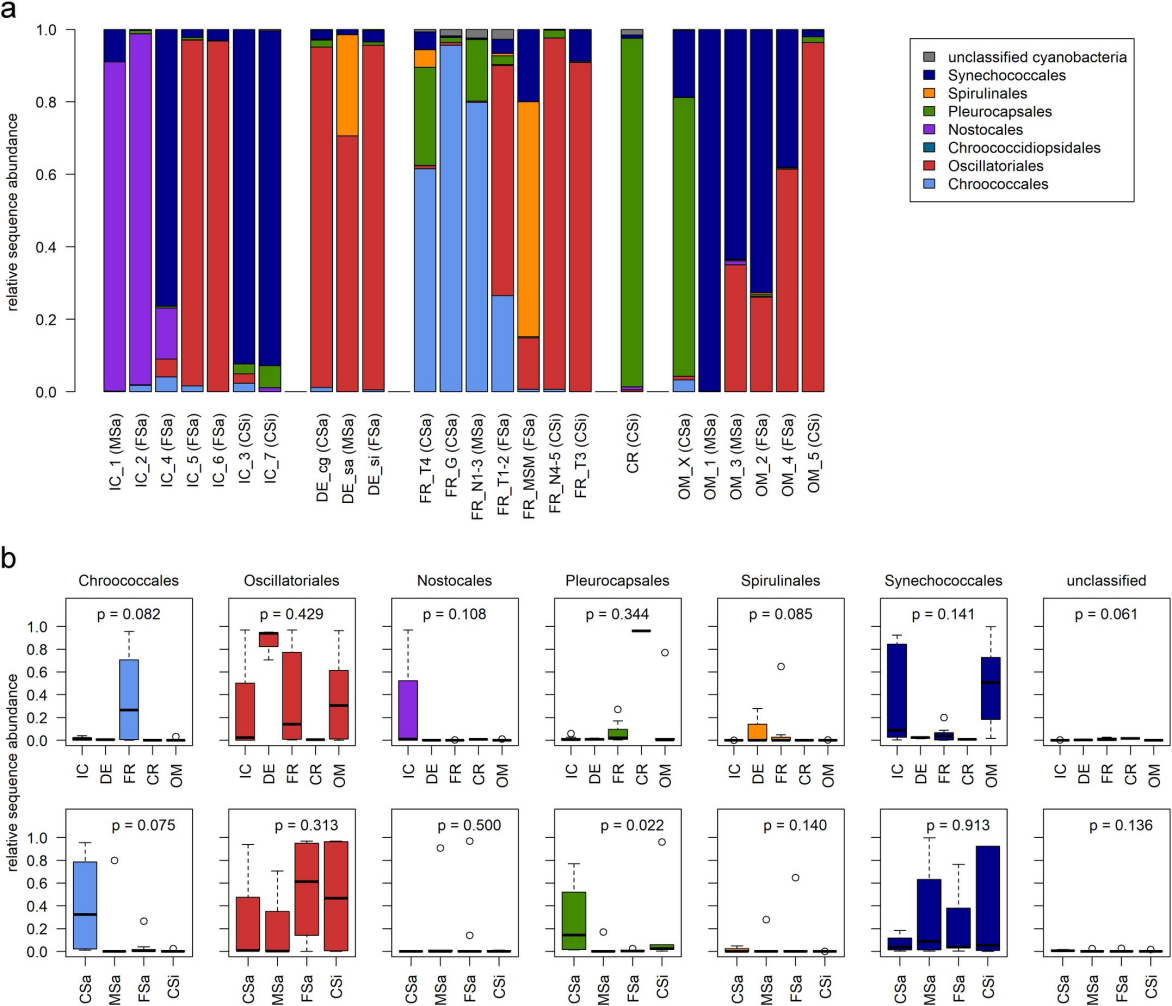
Relative sequence abundance according to taxonomic classification on order level. (a) Relative sequence abundance per sample; samples within each location are sorted according to their mean grain size/sediment type (decreasing); (b) Relative sequence abundance of cyanobacterial orders per location or sediment type; IC = Iceland (n = 7), DE = Germany (n = 3), FR = France (n = 7), CR = Croatia (n = 1), OM = Oman (n = 6); CSa = coarse sand (0.63–2.0mm, n = 4), MSa = medium sand (0.2–0.63mm, n = 5), FSa = fine sand (0.063–0.2mm, n = 9), CSi = coarse silt (0.02–0.063mm, n = 6).

The taxonomic classification on order level revealed a high variability in community composition (based on sequence abundance) within and between locations. However, within some locations (FR, IC, OM) unique grain size patterns were detected (Fig 4A).

Summarized groups per location and sediment type showed differences in community composition and increased abundances of some orders on specific locations or sediment types, however, with wide ranges within each group (Fig 4B). Predominantly unicellular cyanobacteria, such as Chroococcales and Pleurocapsales, were more frequently found on coarse sand sediment, with an exception of the Pleurocapsales-rich Croatian sample that was classified as coarse silt. The order Synechococcales, which includes unicellular and filamentous species, was mainly detected on fine sand to coarse silt in Iceland or on fine to medium sand in Oman. Oscillatoriales dominated samples of all locations and all sediment types, while Spirulinales were mainly found in France and Germany and Nostocales in Iceland, both on fine to medium sand.

**Fig 5:**
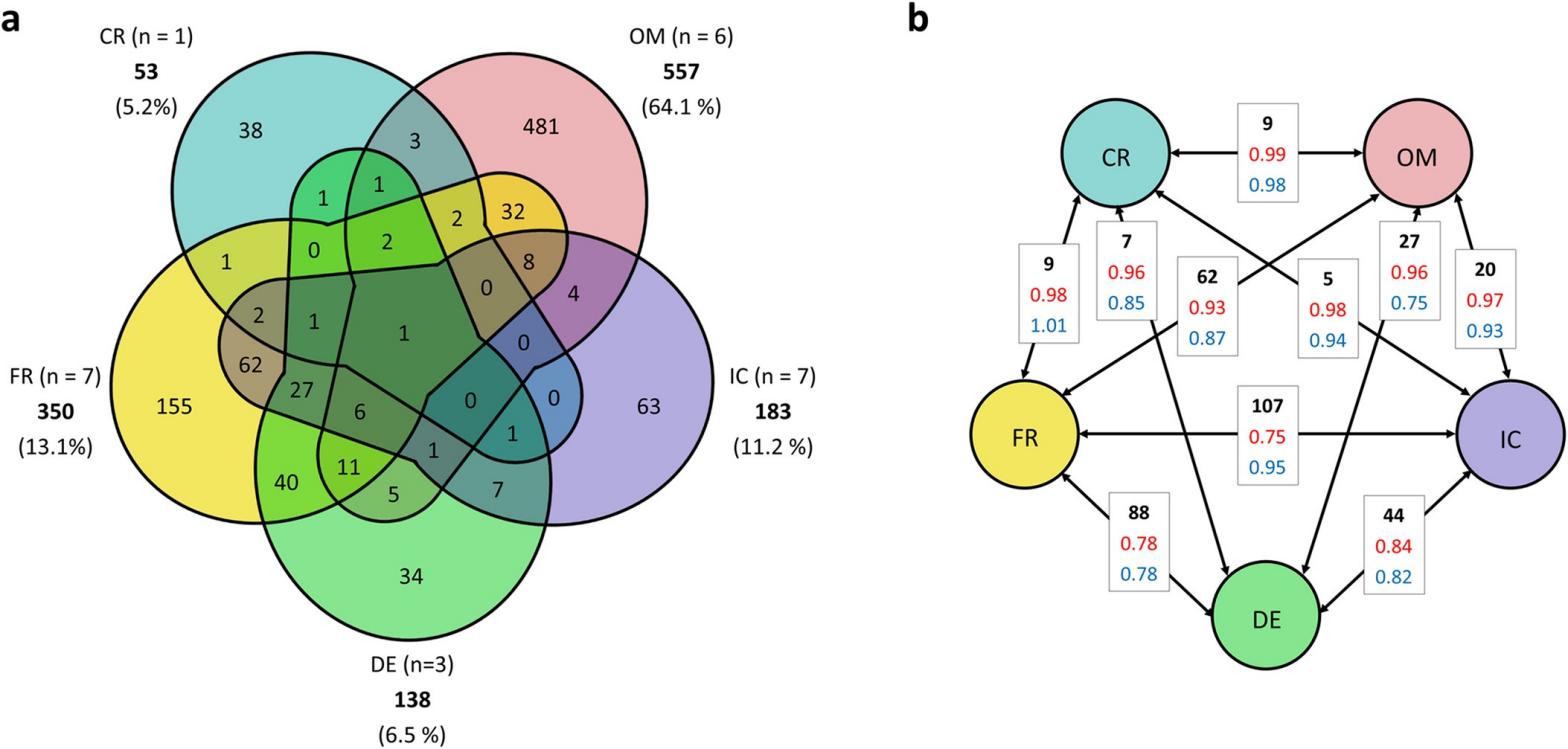
Beta diversity estimates for summarized groups per location. (a) VENN diagram based on shared richness, calculated based on a presence/absence matrix summarizing all samples per location. Number of included samples (n), total OTU numbers and percentage of total sequence numbers were shown per location. (b) Beta diversity estimates of paired location groups showing numbers of shared OTUs (black), Jaccard dissimilarity coefficients (red) and phylogenetic beta diversity (MPD between samples, blue). IC = Iceland, DE = Germany, FR = France, CR = Croatia, OM = Oman.

A large number of OTUs were location specific, with the maximum number present in the Oman samples (481 OTUs). The percentage of location specific OTUs was highest in Oman (86%) and Croatia (72%) and clearly lower in France (44%), Iceland (34%) and Germany (25%). Only one OTU, classified as *Coleofasciculus* (97–100% similarity to a *Coleofasciculus chthonoplastes* sequence (accession number NR_125521.1 in the NCBI database)), was shared by all locations (Fig 5A). Pairwise comparisons of the locations revealed highest numbers of shared OTUs for France—Iceland (107) and France—Germany (88), lowest for Iceland—Oman (20) and Germany—Oman (27), except the single Croatian sample with only 5 to 9 shared OTUs with every other location (Fig 5B). These results were also supported by Jaccard dissimilarity coefficients showing lowest dissimilarity between French—Icelandic (0.75) and French—German (0.78) samples and highest dissimilarity for Croatian samples and all other locations (0.96–0.99), as well as for Oman—Icelandic (0.97) and Oman—German (0.96) samples (Fig 5B). Interestingly, lowest phylogenetic beta diversity was observed for samples from Germany and Oman (0.75) close to that of French and German samples (0.78) (Fig 5B). However, all beta diversity estimates had a rather high dissimilarity/diversity (> 0.75) between the investigated locations.

**Fig 6:**
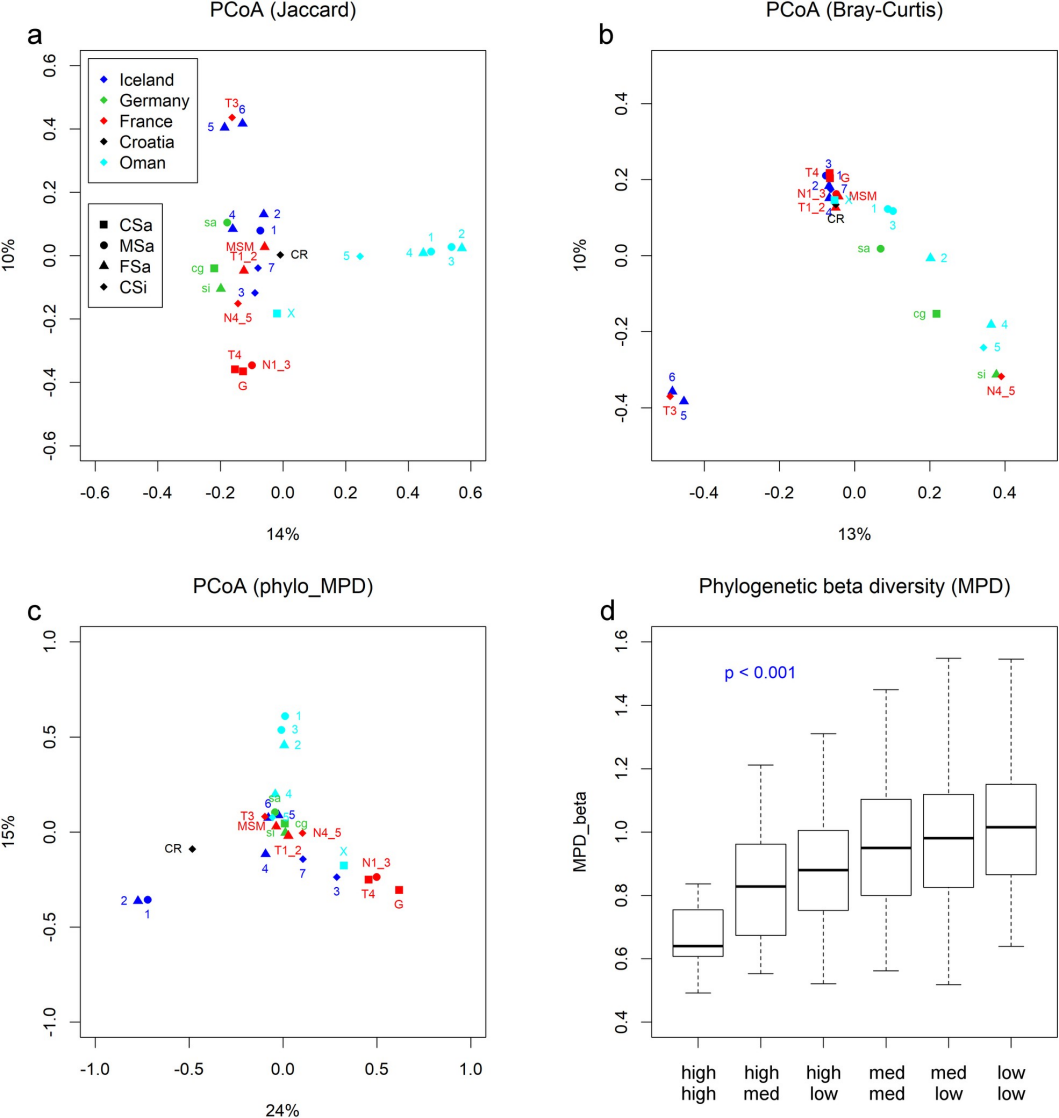
**Dissimilarity patterns revealed by PCoAs (a-c) and phylogenetic beta diversity (MPD) between paired samples according to their stabilization level (d)**. PCoAs were based on Jaccard (a) and Bray-Curtis (b) dissimilarity coefficients and the phylogenetic distance matrix (c) of all representative sequences per OTU (abundance unweighted); the first two axes explain a total variability of 24%, 23% and 39%, in a, b, and c respectively. (d) Kruskal-Wallis tests were performed to identify significant differences of phylogenetic beta diversity estimates (MPD) between samples that were grouped according to their stabilization levels.

Although linear regression analyses between beta diversity estimates and the (logarithmized) linear distances between paired samples revealed significant linear correlations (p < 0.001 (OTU-based), p < 0.01 (phylogenetic) as shown in S4 Fig) and indicate a distance-decay relationship, no clear similarity patterns concerning locations and distances could be verified by PCoAs based on different beta diversity estimates (Fig 6A–6C).

Based on Jaccard dissimilarity coefficients (OTU presence/absence) the PCoA clearly separated the hypersaline samples from Oman (O1-5), three samples from France (T4, G, N1-3) and two Icelandic samples (5, 6) together with one French sample (T3), but the remaining samples built a loose cluster (Fig 6A). PCoA revealed one large cluster that contained most of the samples from France, Iceland, and Croatia and again a separate cluster of two Icelandic (5, 6) and one French sample (T3), based on Bray-Curtis dissimilarity coefficients (including sequence abundance per OTU) (Fig 6B). The hypersaline samples from Oman (1–5) and the German samples (sa, cg, si) formed a tail from the main cluster with the most distant ones (DE_si, OM_4, OM_5) clustering with one French sample (N4-5). Based on the phylogenetic beta diversity (MPD between samples) the PCoA (Fig 6C) revealed one main cluster including samples from all locations. Some hypersaline samples from Oman (1–3), two samples from Iceland (1–2), and the Croatian sample were clearly separated. Three French samples (N1-3, T4, G) formed a tail from the main cluster and were most similar to the less saline sample from Oman (OM_X) and one more Icelandic sample (3).

While all three PCoAs did not reveal clear location or grain size patterns, the stabilization level of the community occurred as potential predictor of at least phylogenetic beta diversity. Comparing the mean pairwise distances between samples (phylogenetic beta diversity) according to their level of stabilization revealed the lowest phylogenetic distances between well-stabilized samples (high—high) and the highest ones between less stabilized ones (low—low) (Fig 6D).

## Discussion

To investigate (global) patterns of cyanobacterial diversity, our study focused here on tidal flats as a highly dynamic habitat type that provides similar environmental conditions at distant sites, but also includes variability in environmental parameters on different spatial scales (e.g. climate, grain size, and nutrients). Thus, tidal flats offer the opportunity to investigate diversity patterns along geographical and ecological gradients within a distinct habitat.

Previous molecular/high-resolution studies on cyanobacterial biogeography and diversity were mainly performed either in island-like environments, like hot springs with strong geographical barriers [23] or in open oceans without or with at least very weak geographical barriers [91; 92]. Opposed to these extremes in terms of putative geographical isolation, tidal flats are delimited habitats, but they are widely distributed along all coastlines without strict boundaries towards the flanking terrestrial and marine habitats. Although each sampling site showed a unique composition of environmental parameters (Table 3, S3 Table), they all have the special tidal character with its periodic fluctuations of environmental conditions in common, which make this habitat an extreme one in terms of wide ranges of diverse abiotic parameters.

Benthic communities from tidal flats and their diversity have been already intensively studied at different locations all over the world [60; 61; 62; 63; 64; 65; 93; 94; 95; 96], but it remains challenging to compare or assemble the results of different studies due to methodological differences. Therefore, it is more than difficult to deduce global patterns of diversity from several datasets of different spatially restricted studies (but see [2; 3]). The novelty of this study is its spatial coverage from the subtropics (Oman, 20°N/58°E) to the subarctic (Iceland, 65°N/22°W), including a set of 24 different samples from five locations combined with the use of a high-throughput sequencing method (Illumina MiSeq) to investigate cyanobacterial diversity patterns in one distinct habitat. So far, there are no comparable studies/datasets available describing cyanobacterial diversity patterns in such a large area based on the same methodologies. Previous studies often used either different, often less resolved methods (e.g. clone libraries, fingerprinting methods, 454 sequencing), and/or were spatially much more restricted (e.g. [24; 49; 50; 51; 53; 62; 63; 97; 98]). In general, most of the studies about global diversity of prokaryotes are restricted to marine and mainly planktonic environments [20; 49; 51; 97], which are likely to facilitate wide distribution ranges of small organisms due to the simple dispersal capabilities in open waters with few geographical barriers. These differences in dispersal capabilities are most probably also one reason why Zinger *et al*. [97] observed a higher similarity with increasing distance between pelagic communities than between benthic ones.

### Latitudinal patterns

Latitudinal diversity gradients as observed here for cyanobacteria in tidal flats (Fig 2) are one of the most widely recognized patterns in ecology [37; 38; 39; 43] since Humboldt & Bonpland [25], Wallace [26; 27] and Darwin [28] described an increased species richness in tropical regions compared to that in temperate ones. There are diverse hypotheses about reasons for these latitudinal differences in species richness. Historically the tropics are supposed to provide more time and opportunity for diversification than temperate regions due to their greater age, historically larger area, and their more stable climate, while the dispersal of organisms out of the tropics is often limited. Therefore, a plentitude of species have accumulated in tropical regions during Earth’s history making them a ‘cradle’ and a ‘museum’ of biodiversity [39; 44]. Due to higher speciation and lower extinction rates caused by environmental/ecological influences as e.g. climatic conditions (e.g. temperature, climate stability), biotic interactions (e.g. competition, predation), ecological opportunity (e.g. tolerance ranges/niche width, dispersal, metabolic rates, generation times) and area effects (e.g. isolation, population size), the diversification rates and evolutionary speed in the tropics are also supposed to be higher compared to temperate regions [44; 99]. However, recent studies have found evidence for an equalization of diversification rates over latitudes and nowadays highest speciation rates in temperate regions [45].

Latitudinal diversity gradients were intensively studied for a wide variety of taxonomic groups in different environments [37; 38; 44; 47; 100; 101; 102]. Clear relationships between the overall diversity on community level, latitude, and temperature were shown to be a result of interfering, distinct biogeographical distribution patterns of different individual taxa across latitudes according to their unique ecological/physiological properties [38; 42]. Therefore, the gradients become clearer with increasing numbers of taxa and habitat types [38; 42; 47], even though they may vary between different habitat types [47]. Although, focusing on cyanobacterial communities in tidal flat regions in this study restricted both the taxonomic coverage and the habitat type, decreasing richness and diversity values (except MPD) from subtropical (20°N) to subarctic regions (65°N) (Fig 2A) verified the hypothesis of a latitudinal diversity gradient for cyanobacteria in tidal flats. This is similar to what has been observed for many other organisms in different habitats [38; 50; 51]. These results support ecological/environmental reasons for the observed latitudinal gradient of cyanobacterial diversity in tidal flats, although additional historical/evolutionary reasons for the present distribution and diversity could not be excluded.

The observed latitudinal diversity gradients (Fig 2) are a clear evidence for biogeographical distribution patterns of cyanobacterial taxa that are not only determined by the presence or absence of taxa within an area, but also by their composition/proportion within a community, which may strongly influence diversity estimates [34]. Microbial diversity is known to be highly variable on temporal scales and associated ecological parameters as shown e.g. for seasonality [34; 62]. Therefore, observed gradients of diversity and biogeographical distribution pattern are snapshots of the current microbial community and may change with time. However, the sampling of microbial communities near the climax state of annual mat development in summer months at the subarctic to subtropical/dry summer locations (IC, DE, FR, CR) compared to perennial microbial mats sampled in winter at the subtropical-arid location (OM) ensure the investigation of a most comparable, complex and diverse microbial community. Our previous seasonal studies at the German location [62] previously demonstrated that alpha diversity of cyanobacterial mat communities in temperate regions is highest in summer to autumn and that it is mainly the composition of the community, that changed during the course of a year, rather than the presence or absence of season-specific taxa.

### Everything is everywhere?–Comparing study sites

Our results obtained from samples collected along geographical and ecological gradients, clearly indicate that not everything is evenly distributed everywhere (Fig 5A). However, our dataset is not sufficient to answer the question if it’s only the environment that selects, or if there are additional drivers of biodiversity, since present-day diversity and distribution patterns are always a result of both historical events and the actual environmental conditions. Hanson *et al*. [34] proposed that (micro-) evolutionary and ecological influences on microbial distribution and diversity are often inseparable, which might also be true here. They predicted that four processes create and maintain biogeographical patterns (and diversity gradients) of microorganisms: selection, drift, dispersal and mutation. These all may be influenced by evolutionary and ecological factors. Considering the tremendous dispersal potential of microorganisms due to their small cell sizes, large population sizes, and short generation times resulting in high dispersal rates, it is highly likely that ecological properties and environmental selection are much more important than overcoming large distances [32; 34; 48; 103; 104]. However, recent transplantation studies of salt marsh sediment communities at the US East Coast demonstrated that transplanted communities tend to maintain their original community structure, despite changed environmental conditions [105].

In line with this, distance-decay effects were shown by significant correlations between beta diversity estimates and (logarithmized) linear distances between samples (S4 Fig), as also observed for bacterial communities in other studies (e.g. along rivers in France and New Zealand [106]). However, such distance gradients are often strongly correlated to ecological gradients such as climate conditions, which potentially also explains the patterns we received. The expected high ecological similarity between France, Germany, and partially Iceland, based on their close geographic location and similar climate conditions, is supported by them having the highest shared richness (107 OTUs FR/IC, 88 OTUs FR/DE, 35 OTUs FR/DE/IC) and lowest dissimilarity values (Jaccard dissimilarity coefficients 0.75–0.84) between the summarized groups of these locations (Fig 5B). Dispersal of microorganisms between France and Germany is facilitated by a direct connection along the coastline. Iceland is at least directly connected via ocean currents, while the distances towards Croatia and Oman along coastlines and via ocean currents as putative dispersal routes, are much longer than the measured linear distances between the sampling sites. Lowest shared richness and similarity between Croatia and all other locations is almost certainly not explained by climate, but more probable by different coastal properties (sandy rock pools vs. sandy beaches), although the lowest sample number for Croatia (1) may have influenced the results as well. Lower shared richness and higher dissimilarity coefficients between the other samples (Fig 5B) were indicative for different ecological conditions and climate, as well as for the large distance to each other, as was observed for Iceland and Croatia, which had only five shared OTUs and a Jaccard dissimilarity coefficient of 0.98.

Beta diversity estimates of the summarized locational groups are highly likely to be influenced by the number of samples per location, that were not even within our study (Table 2). Additional influences on alpha and beta diversity estimates due to the use of different DNA extraction kits and different sequencing runs for the hypersaline samples from Oman and that of all other locations could also not be excluded, since total sequence numbers and the overall richness and diversity in samples from Oman was shown to be highest (Table 2). Additionally, sequence counts during sequence analyses also suggest highest quality (less low quality loss) of Oman sequences (S2 Table).

### Cosmopolitanism vs. endemism/habitat-specificity

Our dataset of 24 samples from 5 locations allowed no general statement about cosmopolitanism or endemism of detected taxa, but instead high amounts of location-/sample-specific OTUs indicated at least (micro-) habitat-specific taxa, since the unique composition of different environmental conditions between the sampled sites clearly influenced community composition.

Despite the common assumption that microorganisms disperse freely around the world without geographical barriers [6; 9; 107], such cosmopolitan distribution patterns could only be verified for one single OTU (classified as *Coleofasciculus chthonoplastes*) across our sampling locations (Fig 5A). This OTU occurred in each location (but not in each sample) and was predominantly abundant/dominant confirming observations of Nemergut *et al*. [108] that cosmopolitan taxa tend to be the abundant members in individual assemblages within one habitat type. The occurrence of shared OTUs between samples from distant locations with differing environmental conditions as the 20 shared OTUs between samples from Oman and Iceland (Fig 5) indicated wide dispersal and wide tolerance ranges of the respective taxa, even if they were not detected at all sampled locations. Shared OTUs that were abundant in samples of different locations could be predominantly classified as common oscillatorialean genera like *Coleofasciculus* and *Lyngbya* (S7 Table), which are known to have wide ecological tolerance and distribution ranges [10; 109]. Proportions of sequences per sample/location within a shared OTU could for sure strongly vary and there were many OTUs dominant at one location, but rare in the other ones. These differences in community composition are again likely to be a result of the environmental conditions and biotic interactions within the community.

The great majority of detected OTUs here were specific for distinct locations (78% of all OTUs, Fig 5A) or even sampling sites (39% of all OTUs). Environmental parameters and their unique composition were supported as important factors for community composition at each location. OTUs specific to single samples or groups makes them unique and often indicates potentially endemic or habitat-specific taxa. These taxa are commonly supposed to have closer tolerance ranges and are therefore dispersal-limited, but specialized or adapted to special/extreme environmental conditions where they are competitively stronger than other taxa. This matched the highest number of specific OTUs (481) at the extreme location in Oman (Fig 5A), which was characterized by hot and hypersaline conditions.

Although most location-specific OTUs were rare members of the community, there were single samples that reached high proportions of sequences within location specific OTUs. For example, up to 73% and 63% of all sequences per sample in samples from Iceland (Otu22, IC_2) and Oman (Otu3, OM_1), respectively. These likely represent the occurrence of specialized (e.g. halophilic) and warm/cold adapted taxa that are able to outcompete less specialized taxa in such extreme locations, as has been previously shown for the study site in Oman [63]. Interestingly, many specific OTUs were affiliated with genera that are usually known to have wide tolerance ranges (e.g. *Coleofasciculus*, *Lyngbya*), which could be either a hind for the development of ecotypes or genetic differences beyond the investigated 16S rRNA gene fragment (insufficient resolution).

Rare members of a community are supposed to be important keystone species and distinguishing marks between communities from different locations and potential indicators for differing environmental influences [3]. They might serve as dormant seedbank and important hidden drivers of ecosystem function improving the metabolic flexibility and physiological tolerance of a community to changing and/or harsh/extreme conditions [1; 110; 111], but their small numbers of individuals make them putatively also more vulnerable to these conditions compared to well-established and abundant taxa [110; 112].

### Abiotic patterns

Temperature and grain size of the inhabited sediment were shown here as most important factors for the spatial distribution of OTUs (Fig 3). Environmental parameters that are strongly correlated to latitude are known as important ecological reasons for latitudinal biodiversity patterns on large spatial/global scales [43; 44]. Such correlations were shown here for temperature and other climatic parameters (S3 Fig). Temperature is commonly assumed to influence speciation rates and natural selection by its impact on metabolic rates and generation times [44; 99]. As previously shown for marine planktonic bacteria from the tropics to the poles [49; 51] and diverse eukaryotic and prokaryotic taxa along elevation gradients [42, 113], temperature was confirmed here as important driver of cyanobacterial biodiversity in tidal flats (Fig 2B, S5 Table). However, although an increasing phylogenetic clustering was previously described for bacterial communities along elevation (temperature) gradients towards mountaintops [114], such a significant impact of temperature on MPD values could not be verified here for cyanobacterial communities along the latitudinal gradient (Fig 2).

Strong linear correlations of climatic and (dissolved) chemical parameters as shown here for salinity and alkalinity (p < 0.001) (S3 Table) were supposed to be mainly caused by climate-related evaporation/desiccation or dilution. The local variability of nutrient concentrations, on the contrary, was rather indicative for putative sampling site specific anthropogenic or environmental influences (e.g. eutrophication) and physiological processes within the respective communities [58;59]. Such (independent) environmental parameters have already been reported by others to modify/shift the temperature-biodiversity relationships (e.g. [113]). We could show this effect in our study for grain size and nutrient concentrations, which were variable across latitudes and additionally influenced diversity (Table 3, S3 Table). However, these parameters are likely to be more important on smaller local scales and for community composition, as shown by Vogt *et al*. [62; 63]. Grain size has previously been reported to be an important factor influencing microbial community development and composition [58; 62; 115] and our results confirmed this parameter as an important driver of cyanobacterial diversity (Fig 3). The observed variability of diversity estimates within locations (Fig 2A) indicated the importance of variable parameters across latitudes for present communities on smaller spatial/local scale. However, there were no consistent (linear) diversity gradients within locations based on the measured parameters in this study as shown e.g. for grain size patterns with inverse trends at different locations (Fig 2V, S5 Fig).

Temporal, especially seasonal dynamics of abiotic and (related) biotic parameters additionally influence the current biodiversity (e.g. [62]). Thus, composition and (functional) complexity of microbial communities are known to change during succession influenced by environmental parameters [58; 61]. The developmental stage and complexity of each community could be roughly estimated according to the degree of stabilization and lamination, which usually increases successively with the number of individuals and members with different physiological properties/niches comprising in the end all major element cycles [61]. However, discontinuities in mat growth and development or even the destruction of benthic microbial mats may be caused by extreme environmental conditions as shown for our study site in Germany during winter season [62]. Furthermore, varying physical factors such as wind and wave activity according to local characteristics of the coastline, and factors like (hyper-) salinity and grain size of the colonized sediment may either facilitate, decelerate, or even change succession and consequently composition of microbial communities [60, 115]. Due to the small number of samples per location in this study and their different ranges of ecological variability within each location, the observed correlations of diversity and abiotic parameters can only be understood as putative trends. In general, a high variability of environmental conditions and sampling sites per location was desired to cover a broad range of intertidal (micro) habitats, but unfortunately it was not possible to sample broad ranges of all measured parameters in each location.

The ability of organisms to cope with or adapt to extreme environmental conditions (e.g. high or low temperatures, hypersalinity), plays an important role for their distribution across latitudes [11]. Due to the potentially limited dispersal of organisms according to their physiological tolerances/plasticity towards environmental conditions, diversity is likely to be restricted at more extreme sites [50; 93]. However, since cyanobacteria are known as important primary producers and pioneer organisms that tolerate a wide range of extreme/harsh environmental conditions, they are expected to show high diversity in extreme environments and early stage mat communities, as has been observed in previous studies [61; 116; 117; 118; 119]. This same trend was shown here for hypersaline samples from Oman and early stage communities from France, Germany and Iceland (Table 2). These results are in line with previous observations by Rothrock and Garcia-Pichel [93], which observed that cyanobacterial diversity along an intertidal desiccation gradient was less impacted than bacterial and archaeal ones, or the study of Pommier *et al*. [49] detecting lowest correlations of cyanobacterial OTU richness to latitude and temperature compared to all other bacterial taxa, and finally the studies of Abed *et al*. [64] and Vogt *et al*. [63], which observed that cyanobacterial diversity even increased along a hypersaline intertidal gradient with increasing distance to lower tidal line. The frequently high tolerance ranges of cyanobacteria for most diverse environmental conditions and their high diversity in extreme environments [120] may be one reason for all these observations.

### Taxonomic patterns and phylogenetic aspects

Taxonomic classification based on 16S rRNA gene fragments as shown here (Fig 4) can only approximate the actual taxonomy, since perfect matches with reference datasets were usually rare. In this study we had only 3 OTUs (<0.5%) with 100% and 88 OTUs (9%) with at least 97% similarity matches within the ‘16S ribosomal RNA sequences (Bacteria and Archaea)’ database. Although, clustering sequences at a 97% similarity level and classifying OTUs on order level in this study (Fig 4A) was appropriate to get an overview of community composition of the samples, one should be aware that gradients of diversity and biogeographical distribution patterns were also shown to be influenced by taxonomic resolution [34].

Contrary trends of OTU-based (Shannon) and phylogeny-based (MPD) diversity estimates across latitudes (Fig 2A) highlighted the importance of phylogenetic/taxonomic information comparing communities and interpreting results. Including phylogenetic distances for diversity estimates allows deeper insights into community structure and function based on the assumption that closely related species/lineages share similar niches (niche conservatism) and their metabolic abilities are conserved [121]. The observed trends of decreasing phylogenetic diversity with increasing mat stabilization (S6 Fig) suggested a shift and restriction of ecological niches of cyanobacteria during succession. The more niches are inherited successively by non-cyanobacterial members of the microbial community, the fewer niches remain for cyanobacterial ones. Moreover, stabilizing compounds within microbial mats may serve as a protective shield against environmental influences cushioning their fluctuations [58; 59; 116]. Thus, the internal physicochemical gradients within the nearly closed small-scaled ecosystem might become more important and are supposed to be more similar across latitudes than the outer environmental conditions. The stronger influences of changing environmental conditions on less stabilized communities could be one reason why they were shown to be phylogenetically much more diverse among the studied locations than well-stabilized communities, which become increasingly similar across latitudes (Fig 6D).

Although annual mean temperature (latitude) and mean grain size were identified as the most important (quantitative) factors influencing sequence abundance (Fig 3), no general latitudinal/temperature related or consistent grain size pattern of taxonomic community composition could be observed on a global scale (Fig 4). However, distinct grain size patterns on smaller spatial scales within each location (Fig 4A) were observed, indicating changing (competitive) success of different taxonomic groups on specific grain sizes dependent on other environmental conditions (e.g. climate). This suggested an overall stronger impact of climate and latitude than granulation of the sediment.

OTUs classified as Oscillatoriales occurred in all locations as dominant member of the community (except Croatia) (Fig 4, S7 Table) confirming earlier studies of Stal *et al*. [58] and Garcia-Pichel *et al*. [10] and indicating a high physiological plasticity and wide distribution range of this order. Their distribution along both RDA axes (Fig 3) indicates that some taxa of the order are rather influenced by changing temperatures whereas others are more influenced by different grain sizes. Oscillatoriales (e.g. *Coleofasciculus*, *Lyngbya*) are known as widespread marine/benthic cyanobacteria and main components of well-stabilized microbial mats [109].

The increased abundance of sequences classified as Spirulinales (Fig 4, S7 Table) matched their description as frequent member of marine coastal communities [109].

The distribution of OTUs classified as Synechococcales was mainly correlated to temperature, as indicated by RDA (Fig 3). This order includes unicellular and filamentous taxa commonly detected in marine and terrestrial environments all over the world [64; 94; 119; 122; 123]. In line with other studies in extreme environments (e.g. [119; 123]) they were here mainly detected in samples from Iceland and Oman in all sediment types with the highest abundances in less developed mats (Fig 4, S7 Table).

Unicellular taxa (Chroococcales, Chroococcidiopsidales, Pleurocapsales, and Synechococcales) are often described in marine and freshwater environments, and are also known to dominate harsh and unfavorable environments from deserts to rocky shores and tidal flats across latitudes [64; 117; 123; 124; 125; 126; 127; 128; 129; 130]. RDA indicated a strong influence of grain size for the distribution of OTUs classified as Chroococcales or Pleurocapsales (Fig 3). As coarse-grained sediment was suggested to be unfavorable for the development of well-stabilized mats with high amounts of filamentous taxa [58], it is not surprising that the unicellular orders reached high sequence abundances in early stage communities on coarse-grained sediment (Fig 4, S7 Table). High production rates for extracellular polymeric substances (EPS) were described for unicellular cyanobacteria [131], privileging them as important autotrophic pioneer organisms at low nutrient sites. Putative input and sedimentation of planktic unicellular taxa from seawater to the benthic communities could be one more reason for their increased abundance in early stage communities.

Although all of the Croatian sequences classified as Pleurocapsales stayed unclassified on genus level (96%), their high sequence abundance (Fig 4, S7 Table) was in line with other studies at this limestone coast [129; 130]. The nearly complete absence of sequences classified as filamentous Oscillatoriales and Nostocales in the well-stabilized Croatian sample is surprising and may be explained by the extraordinary local settings (limestone coast with sandy rock pools) that may favor unicellular species [129; 130]. Also biases during sample preparation could not be excluded, since microscopic investigations (not shown here) detected high amounts of filamentous species (with and without heterocysts) wrapped within thick sheaths, which may have withstood the lysis process during the DNA isolation.

Only two mats from Iceland (IC_1, IC_2) were dominated by OTUs classified as Nostocales (Fig 4, S7 Table), which are known to be able to fix nitrogen and to produce akinetes which help to overcome unfavorable conditions such as frost events [132]. Thus, these taxa are expected to occur at unfavorable and low nutrient sites and also survive at high latitudes with low temperatures and low light intensities in the winter season [132].

In summary our results show that microorganisms’ area of distribution and their distance-decay similarities tend to be significant, but weaker than that for macroorganisms. Our study could confirm finding of Soininen [133] that latitudinal patterns of cyanobacterial distribution are affected by species traits and ecosystem characteristics.

## Conclusions

This study observed cyanobacterial diversity pattern in microbial tidal flat communities from the subtropics to the subarctic along geographical and ecological gradients.

Strong distinctions in alpha diversity and community composition, as well as high dissimilarity values between samples due to high numbers of location-specific taxa (beta diversity), gives the first evidence that a true cosmopolitan distribution of cyanobacterial communities does not exist. However, the increased phylogenetic similarity of well-stabilized samples across latitudes supported at least wide distribution patterns of a few closely related cyanobacterial taxa that are predominantly found in well-stabilized microbial mat systems.

Latitudinal diversity gradients of cyanobacterial communities in tidal flats similar to those known from plants and animals were identified here for OTU- and phylogeny-based alpha diversity estimates (especially richness values). Latitude and related environmental parameters (e.g. climate, temperature) were supported as important drivers of diversity on global scale, while environmental parameters that are not correlated to latitude/climate (e.g. grain size, nutrients) were shown to be more important on a local scale. However, these local patterns were not consistent across latitudes or different locations. Environmental parameters such as temperature/latitude and grain size were also shown to be the most important factors influencing the distribution of detected cyanobacterial taxa.

So far, our dataset of 24 samples from 5 locations provides the best spatial coverage within a single study using high-throughput sequencing methods. However, further studies including more samples and locations could help to generalize the results. Higher coverage of similar ecological conditions at distant sites and different ecological conditions at nearby sites could give more convincing inferences about biogeography of specific cyanobacteria taxa.

## Supporting information

S1 Table Overview of sampling sites with sampling date, location and geographic position. (PDF)

S2 Table Sequence counts (nseqs) during sequence analysis, OTU counts of the final dataset, and normalized values based on the final dataset. Sequence and OTU numbers were shown per sample. Percentage of raw data was calculated for averaged sequence numbers per location and for summarized sequence numbers of the complete dataset.
(PDF)

S3 Table Abiotic parameters of the sampling sites. Climate data: https://eosweb.larc.nasa.gov/sse/ (averaged monthly values from 1983–2005), accessed 21.09.2017; water temperatures (estimated from values of nearby locations): www.seatemperature.org, accessed 02.08.2017. Salinity, total alkalinity (TA), and nutrient concentrations (NH_4_, NO_2_, NO_3_, NO_X_, PO_4_) in sea (S) and pore (P) water. No (pore) water samples could be obtained for samples CR and OM_1.
(PDF)

S4 Table Significance values (p-values) of linear regression analyses between different abiotic parameters. Salinity (sal), total alkalinity (TA) and nutrient concentrations (NH_4_, NO_2_, NO_3_, NO_X_, PO_4_) in pore (P) and sea (S) water samples. cor = significant (p < 0.05) negative (neg) or positive (pos) linear correlation.
(PDF)

S5 Table Significance values (p-values) of linear regression analyses between abiotic parameters and alpha diversity. Diversity was described as observed richness (sobs), Shannon diversity index (shannon), Faith’s phylogenetic diversity (PD) and the abundance weighted mean pairwise distance (MPD) based on the Maximum likelihood tree of representative sequences. Salinity (sal), total alkalinity (TA) and nutrient concentrations (NH_4_, NO_2_, NO_3_, NO_X_, PO_4_) in pore (P) and sea (S) water samples. cor = significant (p < 0.05) negative (neg) or positive (pos) linear correlation.
(PDF)

S6 Table Effect summary of selected environmental parameters used for RDA. RDA based on sequence abundances per OTU and selected standardized environmental parameters(independent, non- or less correlated). Sequence abundance per sample and OTU (+ 0.5 to avoid zero values) were centered log-ratio (clr) transformed. Total and pure effects of selected environmental parameters were calculated using permuRDAv1.6.R (https://github.com/chassenr/ARISA).
(PDF)

S7 Table Taxonomic classification and relative sequence abundances of each detected taxon per sample on order and genus level. Iceland (IC), Germany (DE), France (FR), Croatia (CR), Oman (OM). Color gradient indicate high abundance (red) to low abundance (white), and absence (grey) of taxa within each sample.
(PDF)

S1 Fig OTU- and phylogeny-based alpha diversity. Boxplots of sequence numbers, alpha diversity estimated and percentage of rare OTUs per location. OTU-based diversity is described as observed richness and Shannon diversity index, phylogeny-based diversity is described as Faith’s phylogenetic diversity (PD) and the abundance weighted mean pairwise distance (MPD) based on the Maximum likelihood tree of representative sequences per OTU; rare OTUs contain less than 0.1% of all sequences per sample; CR = Croatia, FR = France, DE = Germany, IC = Iceland, OM = Oman, p-values of Kruskal-Wallis tests were shown.
(TIF)

S2 Fig Rarefaction curves for observed OTUs of all samples per location. Rarefaction curves were calculated with 1000 randomizations based on all quality checked sequences per sample using a re-sampling without replacement approach. Colors code locations: dark blue = Iceland (IC), red = France (FR), black = Croatia (CR), green = Germany (DE), light blue = Oman (OM).
(TIF)

S3 Fig Linear regression analyses of climatic parameters and mean grain size in relation to latitude. Colors code locations: dark blue = Iceland (IC), red = France (FR), black = Croatia (CR), green = Germany (DE), light blue = Oman (OM).
(TIF)

S4 Fig Linear regression analyses of beta diversity estimates and distances between sample pairs. Beta diversity is described as shared richness, Jaccard and Bray-Curtis dissimilarity coefficients (OTU-based), and phylogenetic beta diversity (MPD), distance was previously log transformed.
(TIF)

S5 Fig Linear correlations between Shannon diversity estimates and mean grain sizes within locations. Colors code locations: dark blue = Iceland (IC), red = France (FR), black = Croatia (CR), green = Germany (DE), light blue = Oman (OM).
(TIF)

S6 Fig Relationship between stabilization level and mean grain size as well as diversity estimates. Diversity was described as observed richness (sobs), Shannon diversity index (shannon), Faith’s phylogenetic diversity (PD), and the abundance weighted mean pairwise distance (MPD) based on the Maximum likelihood tree of representative sequences. The stabilization level of all samples was qualitatively classified into three classes: high (well-stabilized and laminated microbial mats, that can be peeled off the sediment in large pieces (> 25 cm^2^)), medium (slightly stabilized sediment without clear laminations, that cannot be peeled off the sediment in large, but smaller pieces), and low (lose sediment without notably amounts of stabilizing compounds). p-values of Kruskal-Wallis tests were shown.
(TIF)
