# Supplementary material for: Latitudinal gradient of cyanobacterial diversity in tidal flats
Source: PLoS One. 2019 Nov 13;14(11):e0224444. doi: 10.1371/journal.pone.0224444 (PMC6853291; doi:10.1371/journal.pone.0224444)
Supplement: S1 Table — (PDF) [file pone.0224444.s001.pdf]

S1 Table. Overview of sampling sites with sampling date, location and geographic position.

Location	sample	sampling date	sampling site		GPS coordinates	
Iceland	IC_1	31.07.2015	Vik	Atlantic Ocean	63°24'11.5"N	19°08'01.2"W
	IC_2	31.07.2015	Vik	Atlantic Ocean	63°24'25.6''N	19°03'41.9''W
	IC_3	02.08.2015	Borgarnes	Atlantic Ocean	64°33'07.6"N	21°54'34.9"W
	IC_4	02.08.2015	Hofn	Atlantic Ocean	64°27'48.5"N	21°59'14.8"W
	IC_5	04.08.2015	Mosfellsbær	Atlantic Ocean	64°10'56.3"N	21°42'45.8"W
	IC_6	06.08.2015	Alftanes	Atlantic Ocean	64°05'31.3''N	22°00'19.5''W
	IC_7	07.08.2015	Vestmannaeyjar	Atlantic Ocean	63°26'26.3''N	20°14'05.2''W
Germany	DE_cg	30.07.2014	Hooksiel	North Sea	53°38'34.7"N	08°03'43.5"E
	DE_sa	30.07.2014	Hooksiel	North Sea	53°38'34.9"N	08°03'43.8"E
	DE_si	30.07.2014	Hooksiel	North Sea	53°38'35.4"N	08°03'43.2"E
France	FR_N1-3	13.07.2015	Île de Noirmoutier	Atlantic Ocean	46°53'32.3''N	02°08'05.8''W
	FR_N4-5	13.07.2015	Île de Noirmoutier	Atlantic Ocean	46°54'08.5''N	02°09'01.4''W
	FR_T1-2	16.07.2015	La Trinité-sur-Mer	Atlantic Ocean	47°34'58.2''N	03°00'50.1''W
	FR_T3	16.07.2015	La Trinité-sur-Mer	Atlantic Ocean	47°35'07.7''N	02°59'52.0''W
	FR_T4	18.07.2015	La Trinité-sur-Mer	Atlantic Ocean	47°34'41.7''N	03°02'37.0''W
	FR_G	17.07.2015	Gâvres	Atlantic Ocean	47°41'22.1''N	03°17'46.2''W
	FR_MSM	18.07.2015	Le Mont-Saint-Michel	Atlantic Ocean	48°37'60.0''N	01°30'41.6''W
Croatia	CR	29.07.2013	Sakarun	Adriatic Sea	44°07'45.0''N	14°51'52.3''E
Oman	OM_1	20.12.2014	Masirah	Gulf of Oman	20°46'35.5"N	58°37'59.2"E
	OM_2	20.12.2014	Masirah	Gulf of Oman	20°46'24.3"N	58°38'10.1"E
	OM_3	20.12.2014	Masirah	Gulf of Oman	20°46'01.5"N	58°38'30.8"E
	OM_4	20.12.2014	Masirah	Gulf of Oman	20°45'38.4"N	58°38'52.2"E
	OM_5	20.12.2014	Masirah	Gulf of Oman	20°44'24.7"N	58°40'22.0"E
	OM_X	20.12.2014	Filim	Gulf of Oman	20°35'37.9"N	58°15'40.5"E
