# Supplementary material for: Latitudinal gradient of cyanobacterial diversity in tidal flats
Source: PLoS One. 2019 Nov 13;14(11):e0224444. doi: 10.1371/journal.pone.0224444 (PMC6853291; doi:10.1371/journal.pone.0224444)
Supplement: S2 Table — Sequence and OTU numbers were shown per sample. Percentage of raw data was calculated for averaged sequence numbers per location and for summarized sequence numbers of the complete dataset. (PDF) [file pone.0224444.s002.pdf]

S2 Table. **Sequence counts (nseqs) during sequence analysis, OTU counts of the final dataset, and normalized values based on the final dataset.**

location	group	nseqs	nseqs	nseqs	nseqs	OTUs	nseqs	OTUs
		raw data	quality checked	Chimera checked	final		normalized	
Iceland	IC_1	12679	7158	6018	**5823**	**16**	1862	12
	IC_2	10599	6099	5267	**5053**	**25**	1861	22
	IC_4	23033	9233	4702	**1863**	**37**	1863	37
	IC_5	62899	36914	16714	**15570**	**108**	1854	81
	IC_6	13588	7733	6644	**6432**	**48**	1859	41
	IC_3	23602	12838	6674	**3607**	**20**	1865	20
	IC_7	32816	16966	11351	**9131**	**23**	1862	20
	**ave_IC**	**25602**	**13849**	**8196**	**6783**	**40**	**1861**	**33**
	**% of raw**	**100**	**54**	**32**	**26**		**7**	
Germany	DE_cg	63314	37197	13564	**12592**	**113**	1857	82
	DE_sa	51516	25543	7549	**6962**	**55**	1859	45
	DE_si	32413	18727	8810	**8087**	**73**	1861	55
	**ave_DE**	**49081**	**27156**	**9974**	**9214**	**80**	**1859**	**61**
	**% of raw**	**100**	**55**	**20**	**19**		**4**	
France	FR_T4	52114	29243	9761	**6883**	**124**	1859	97
	FR_G	13819	8086	2646	**2228**	**56**	1869	56
	FR_N1_3	19515	8450	4811	**3558**	**60**	1875	60
	FR_T1_2	50230	24921	11952	**9678**	**76**	1864	63
	FR_MSM	67748	40052	20335	**7502**	**36**	1862	34
	FR_N4_5	36603	20189	12424	**11300**	**63**	1860	47
	FR_T3	39281	23419	15823	**14648**	**90**	1855	53
	**ave_FR**	**39901**	**22051**	**11107**	**7971**	**72**	**1863**	**59**
	**% of raw**	**100**	**55**	**28**	**20**		**5**	
Croatia	CR	43357	25039	22920	**22063**	**53**	1858	43
	**ave_CR**	**43357**	**25039**	**22920**	**22063**	**53**	**1858**	**43**
	**% of raw**	**100**	**58**	**53**	**51**		**4**	
Oman	OM_X	151083	121860	76069	**71549**	**174**	1856	103
	OM_1	106687	84501	62858	**60953**	**154**	1851	57
	OM_3	61891	46925	25131	**23074**	**204**	1848	125
	OM_2	52748	38910	15380	**12647**	**276**	1851	180
	OM_4	114707	87827	60722	**57341**	**179**	1848	74
	OM_5	91860	69229	49973	**47186**	**135**	1861	73
	**ave_OM**	**96496**	**74875**	**48356**	**45458**	**187**	**1853**	**102**
	**% of raw**	**100**	**78**	**50**	**47**		**2**	
**total numbers**		**1228102**	**807059**	**478098**	**425730**	**989**	**44620**	**889**
**% of raw data**		**100**	**66**	**39**	**35**		**4**	

Sequence and OTU numbers were shown per sample. Percentage of raw data was calculated for averaged sequence numbers per location and for summarized sequence numbers of the complete dataset.
