# Supplementary material for: Latitudinal gradient of cyanobacterial diversity in tidal flats
Source: PLoS One. 2019 Nov 13;14(11):e0224444. doi: 10.1371/journal.pone.0224444 (PMC6853291; doi:10.1371/journal.pone.0224444)
Supplement: S3 Table — Climate data: https://eosweb.larc.nasa.gov/sse/ (averaged monthly values from 1983–2005), accessed 21.09.2017; water temperatures (estimated from values of nearby locations): www.seatemperature.org, accessed 02.08.2017. Salinity, total alkalinity (TA), and nutrient concentrations (NH4, NO2, NO3, NOX, PO4) in sea (S) and pore (P) water. No (pore) water samples could be obtained for samples CR and OM_1. (PDF) [file pone.0224444.s003.pdf]

S3 Table. Abiotic parameters of the sampling sites.

location	sample	annual mean air temperature [°C]	annual mean water temperature [°C]	annual mean precipitation [mm/day]	vegetation period [month > 10°C]	grain size [µm]	Salinity [%]		TA [mM]		NH_4 [µM]		NO_2 [µM]		NO_3 [µM]		NO_X [µM]		PO_4 [µM]	
							P	S	P	S	P	S	P	S	P	S	P	S	P	S
Iceland	IC_1	4.5	8.9	4.3	0	516.5	0.2	0.2	0.0	0.4	7.4	7.8	0.9	0.0	NA	26.3	NA	26.3	0.0	0.0
	IC_2	4.5	8.9	4.3	0	168.7	3.1	3.1	0.0	1.8	4.2	2.6	0.4	0.0	NA	11.1	NA	11.1	0.1	0.0
	IC_4	3.0	7.0	4.1	1	98.8	3.2	3.2	1.7	1.8	2.2	3.5	0.0	0.0	2.2	8.6	2.2	8.6	0.0	0.0
	IC_5	3.0	7.7	4.1	1	76.3	2.2	2.5	1.3	1.4	3.5	7.2	0.0	0.0	7.7	9.0	7.7	9.0	0.0	0.0
	IC_6	4.3	7.5	3.9	1	75.0	3.8	3.9	1.5	1.9	2.3	16.5	0.0	0.0	0.4	2.5	0.4	2.5	2.0	0.1
	IC_3	3.0	7.0	4.1	1	51.0	0.8	0.9	0.0	0.3	3.5	4.8	0.0	0.0	5.4	8.6	5.4	8.6	0.3	0.0
	IC_7	5.0	8.9	4.2	2	33.2	3.1	3.1	2.3	2.2	1.8	6.1	0.0	0.6	5.0	7.1	5.0	7.7	2.5	2.2
Germany	DE_cg	9.6	11.2	2.9	6	778.7	4.5	3.5	6.8	2.9	46.6	19.6	1.6	1.0	2.9	3.4	4.5	4.5	33.5	3.2
	DE_sa	9.6	11.2	2.9	6	474.2	4.9	3.5	9.7	2.9	290.8	19.6	3.0	1.0	0.3	3.4	2.2	4.5	46.7	3.2
	DE_si	9.6	11.2	2.9	6	101.9	4.6	3.5	5.1	2.9	53.6	19.6	0.0	1.0	0.0	3.4	0.0	4.5	30.8	3.2
France	FR_T4	12.9	14.1	2.3	8	769.9	3.8	3.7	2.6	2.1	14.0	0.0	0.0	0.0	4.3	0.0	4.3	0.0	2.8	0.4
	FR_G	12.9	14.1	2.3	8	634.6	3.9	3.7	2.2	2.1	75.2	0.0	0.0	0.0	0.0	0.0	0.0	0.0	7.1	0.9
	FR_N1-3	13.8	14.4	2.2	8	406.9	3.8	3.7	2.8	2.1	19.4	6.1	0.0	0.3	0.0	1.0	0.0	1.3	1.4	1.1
	FR_T1-2	12.9	14.1	2.3	8	153.6	3.6	3.6	2.7	2.1	18.1	0.0	0.0	0.0	0.1	0.3	0.1	0.3	1.1	0.6
	FR_MSM	11.9	13.3	2.2	6	128.4	4.6	3.7	3.1	1.1	50.2	0.0	1.6	0.0	0.0	1.0	1.0	1.0	2.6	0.4
	FR_N4-5	13.8	14.4	2.2	8	49.3	4.6	3.8	4.3	2.2	35.9	0.0	0.0	0.0	0.1	0.0	0.1	0.0	9.0	0.5
	FR_T3	12.6	14.1	2.3	7	33.5	4.2	3.7	2.9	2.1	42.3	0.0	0.0	0.0	0.0	3.0	0.0	3.0	1.2	0.6
Croatia	CR	13.4	18.6	2.8	7	62.1	NA	3.9	NA	NA	NA	NA	NA	NA	NA	NA	NA	NA	NA	NA
Oman	OM_X	27.7	27.3	0.3	12	790.7	5.4	5.4	3.2	3.0	0.0	1.6	0.0	0.0	0.7	0.0	0.7	0.0	2.6	0.4
	OM_1	27.7	27.3	0.3	12	473.0	NA	18.0	NA	3.6	NA	34.1	NA	0.3	NA	2.3	NA	2.6	NA	2.0
	OM_3	27.7	27.3	0.3	12	352.3	19.0	18.0	3.7	3.6	30.3	34.1	0.3	0.3	1.6	2.3	1.9	2.6	1.8	2.0
	OM_2	27.7	27.3	0.3	12	193.9	19.0	18.0	3.7	3.6	40.4	34.1	0.3	0.3	5.2	2.3	5.5	2.6	2.7	2.0
	OM_4	27.7	27.3	0.3	12	151.2	18.0	18.0	3.6	3.6	34.4	34.1	0.3	0.3	2.3	2.3	2.6	2.6	2.7	2.0
	OM_5	27.7	27.3	0.3	12	58.7	18.0	18.0	3.6	3.6	35.2	34.1	0.3	0.3	3.0	2.3	3.3	2.6	2.1	2.0

Climate data: https://eosweb.larc.nasa.gov/sse/ (averaged monthly values from 1983-2005), accessed 21.09.2017; water temperatures (estimated from values of nearby locations): www.seatemperature.org, accessed 02.08.2017. Salinity, total alkalinity (TA), and nutrient concentrations (NH_4, NO_2, NO_3, NO_X, PO_4) in sea (S) and pore (P) water**.** No (pore) water samples could be obtained for samples CR and OM_1.
