# Supplementary material for: Latitudinal gradient of cyanobacterial diversity in tidal flats
Source: PLoS One. 2019 Nov 13;14(11):e0224444. doi: 10.1371/journal.pone.0224444 (PMC6853291; doi:10.1371/journal.pone.0224444)
Supplement: S4 Table — Salinity (sal), total alkalinity (TA) and nutrient concentrations (NH4, NO2, NO3, NOX, PO4) in pore (P) and sea (S) water samples. cor = significant (p < 0.05) negative (neg) or positive (pos) linear correlation. (PDF) [file pone.0224444.s004.pdf]

S4 Table. Significance values (p-values) of linear regression analyses between different abiotic parameters.

	latitude	cor	annual mean air temperature	cor	annual mean water temp	cor	annual mean precipitation	cor	grain size	cor
**sal_S**	**< 0.001**	neg	**< 0.001**	pos	**< 0.001**	pos	**< 0.001**	neg	**0.883**	
**sal_P**	**< 0.001**	neg	**< 0.001**	pos	**< 0.001**	pos	**< 0.001**	neg	**0.630**	
**TA_S**	**< 0.001**	neg	**< 0.001**	pos	**< 0.001**	pos	**< 0.001**	neg	**0.431**	
**TA_P**	**0.167**		**0.165**		**0.260**		**0.087**		**0.226**	
**NH_4_S**	**0.001**	neg	**0.001**	pos	**< 0.001**	pos	**0.004**	neg	**0.893**	
**NH_4_P**	**0.848**		**0.853**		**0.993**		**0.674**		**0.393**	
**NO_2_S**	**0.832**		**0.805**		**0.901**		**0.775**		**0.463**	
**NO_2_P**	**0.750**		**0.741**		**0.674**		**0.851**		**0.192**	
**NO_3_S**	**0.016**	pos	**0.017**	neg	**0.038**	neg	**0.003**	pos	**0.720**	
**NO_3_P**	**0.614**		**0.578**		**0.774**		**0.376**		**0.608**	
**NO_X_S**	**0.016**	pos	**0.016**	neg	**0.037**	neg	**0.003**	pos	**0.756**	
**NO_X_P**	**0.616**		**0.577**		**0.754**		**0.398**		**0.855**	
**PO_4_S**	**0.089**		**0.082**		**0.109**		**0.076**		**0.463**	
**PO_4_P**	**0.653**		**0.658**		**0.511**		**0.831**		**0.177**	

Salinity (sal), total alkalinity (TA) and nutrient concentrations (NH_4, NO_2, NO_3, NO_X, PO_4) in pore (P) and sea (S) water samples. cor = significant ($p < 0.05$) negative (neg) or positive (pos) linear correlation.
