# Supplementary material for: Latitudinal gradient of cyanobacterial diversity in tidal flats
Source: PLoS One. 2019 Nov 13;14(11):e0224444. doi: 10.1371/journal.pone.0224444 (PMC6853291; doi:10.1371/journal.pone.0224444)
Supplement: S5 Table — Diversity was described as observed richness (sobs), Shannon diversity index (shannon), Faith’s phylogenetic diversity (PD) and the abundance weighted mean pairwise distance (MPD) based on the Maximum likelihood tree of representative sequences. Salinity (sal), total alkalinity (TA) and nutrient concentrations (NH4, NO2, NO3, NOX, PO4) in pore (P) and sea (S) water samples. cor = significant (p < 0.05) negative (neg) or positive (pos) linear correlation. (PDF) [file pone.0224444.s005.pdf]

S5 Table. Significance values (p-values) of linear regression analyses between abiotic parameters and alpha-diversity.

	sobs	cor	shannon	cor	PD	cor	MPD	cor
**latitude**	**< 0.001**	neg	**0.011**	neg	**< 0.001**	neg	**0.552**	
**annual mean air temperature**	**< 0.001**	pos	**0.011**	pos	**< 0.001**	pos	**0.534**	
**annual mean water temperature**	**< 0.001**	pos	**0.019**	pos	**< 0.001**	pos	**0.447**	
**annual mean precipitation**	**< 0.001**	neg	**0.005**	neg	**< 0.001**	neg	**0.683**	
**veg. period > 10°C**	**< 0.001**	pos	**0.006**	pos	**< 0.001**	pos	**0.727**	
**grain size**	**0.263**		**0.211**		**0.175**		**0.109**	
**sal_S**	**< 0.001**	pos	**0.024**	pos	**< 0.001**	pos	**0.386**	
**sal_P**	**< 0.001**	pos	**0.009**	pos	**< 0.001**	pos	**0.509**	
**TA_S**	**< 0.001**	pos	**0.078**		**< 0.001**	pos	**0.109**	
**TA_P**	**0.209**		**0.668**		**0.343**		**0.450**	
**NH_4_S**	**< 0.001**	pos	**0.434**		**0.016**	pos	**0.136**	
**NH_4_P**	**0.889**		**0.753**		**0.502**		**0.833**	
**NO_2_S**	**0.638**		**0.477**		**0.933**		**0.252**	
**NO_2_P**	**0.620**		**0.477**		**0.391**		**0.902**	
**NO_3_S**	**0.054**		**0.017**	neg	**0.032**	neg	**0.983**	
**NO_3_P**	**0.305**		**0.069**		**0.339**		**0.050**	
**NO_X_S**	**0.057**		**0.014**	neg	**0.032**	neg	**0.960**	
**NO_X_P**	**0.302**		**0.086**		**0.376**		**0.058**	
**PO_4_S**	**0.092**		**0.833**		**0.260**		**0.176**	
**PO_4_P**	**0.795**		**0.272**		**0.618**		**0.425**	

Diversity was described as observed richness (sobs), Shannon diversity index (shannon), Faith's phylogenetic diversity (PD) and the abundance weighted mean pairwise distance (MPD) based on the Maximum likelihood tree of representative sequences. Salinity (sal), total alkalinity (TA) and nutrient concentrations (NH_4, NO_2, NO_3, NO_X, PO_4) in pore (P) and sea (S) water samples. cor = significant ($p < 0.05$) negative (neg) or positive (pos) linear correlation.
