# Supplementary material for: Latitudinal gradient of cyanobacterial diversity in tidal flats
Source: PLoS One. 2019 Nov 13;14(11):e0224444. doi: 10.1371/journal.pone.0224444 (PMC6853291; doi:10.1371/journal.pone.0224444)
Supplement: S6 Table — RDA based on sequence abundances per OTU and selected standardized environmental parameters(independent, non- or less correlated). Sequence abundance per sample and OTU (+ 0.5 to avoid zero values) were centered log-ratio (clr) transformed. Total and pure effects of selected environmental parameters were calculated using permuRDAv1.6.R (https://github.com/chassenr/ARISA). (PDF) [file pone.0224444.s006.pdf]

S6 Table. Effect summary of selected environmental parameters used for RDA.

Parameter	Effect	R_{adj}	F	p
**mean_grain_size**	pure	0.036	1.890	0.009
**mean_grain_size**	total	0.023	1.551	0.044
**mean_temp_an**	pure	0.098	3.459	0.001
**mean_temp_an**	total	0.086	3.166	0.001
**NH4_P**	total	-0.016	0.640	0.598
**NOX_P**	total	-0.010	0.782	0.750
**PO4_P**	total	0.010	1.242	0.205
**all**	pure	0.122	2.592	0.001

RDA based on sequence abundances per OTU and selected standardized environmental parameters (independent, non- or less correlated). Sequence abundance per sample and OTU (+ 0.5 to avoid zero values) were centered log-ratio (clr) transformed. Total and pure effects of selected environmental parameters were calculated using permuRDAv1.6.R (https://github.com/chassenr/ARISA).
