# Supplementary material for: Latitudinal gradient of cyanobacterial diversity in tidal flats
Source: PLoS One. 2019 Nov 13;14(11):e0224444. doi: 10.1371/journal.pone.0224444 (PMC6853291; doi:10.1371/journal.pone.0224444)
Supplement: S7 Table — Iceland (IC), Germany (DE), France (FR), Croatia (CR), Oman (OM). Color gradient indicate high abundance (red) to low abundance (white), and absence (grey) of taxa within each sample. (PDF) [file pone.0224444.s007.pdf]

S7 Table. Taxonomic classification and relative sequence abundances of each detected taxon per sample on order and genus level.

	IC							DE			FR							CR	OM					
	1	2	4	5	6	3	7	cg	sa	si	T4	G	N1-3	T1-2	MSM	N4-5	T3		X	1	3	2	4	5
**Chroococcales**	0.00	0.02	0.04	0.02	0.00	0.02	0.00	0.01	0.00	0.01	0.62	0.96	0.80	0.27	0.01	0.01	0.00	0.00	0.03	0.00	0.00	0.00	0.00	0.00
**Oscillatoriales**	0.00	0.00	0.05	0.95	0.97	0.03	0.00	0.94	0.71	0.95	0.01	0.01	0.00	0.63	0.14	0.97	0.91	0.01	0.01	0.00	0.35	0.26	0.61	0.96
**Chroococcidiopsidales**	0.00	0.00	0.00	0.00	0.00	0.00	0.00	0.00	0.00	0.00	0.00	0.00	0.00	0.00	0.00	0.00	0.00	0.00	0.00	0.00	0.00	0.00	0.00	0.00
**Nostocales**	0.91	0.97	0.14	0.00	0.00	0.00	0.01	0.00	0.00	0.00	0.00	0.00	0.00	0.00	0.00	0.00	0.00	0.01	0.00	0.00	0.01	0.00	0.00	0.00
**Pleurocapsales**	0.00	0.01	0.00	0.01	0.00	0.03	0.06	0.02	0.00	0.01	0.27	0.01	0.17	0.02	0.00	0.02	0.00	0.96	0.77	0.00	0.00	0.00	0.00	0.02
**Spirulinales**	0.00	0.00	0.00	0.00	0.00	0.00	0.00	0.00	0.28	0.00	0.05	0.00	0.00	0.01	0.65	0.00	0.00	0.00	0.00	0.00	0.00	0.00	0.00	0.00
**Synechococcales**	0.09	0.00	0.76	0.02	0.03	0.92	0.92	0.02	0.01	0.03	0.05	0.00	0.00	0.04	0.20	0.00	0.09	0.01	0.18	1.00	0.63	0.73	0.38	0.02
**unclassified Cyanobacteria**	0.00	0.00	0.00	0.00	0.00	0.00	0.00	0.00	0.00	0.00	0.01	0.02	0.02	0.03	0.00	0.00	0.00	0.02	0.00	0.00	0.00	0.00	0.00	0.00

		IC							DE			FR							CR	OM					
		1	2	4	5	6	3	7	cg	sa	si	T4	G	N1-3	T1-2	MSM	N4-5	T3		X	1	3	2	4	5
**Chroococcales**	**Crocosphaera**	0.00	0.00	0.00	0.00	0.00	0.00	0.00	0.00	0.00	0.00	0.04	0.03	0.00	0.00	0.00	0.00	0.00	0.00	0.00	0.00	0.00	0.00	0.00	0.00
	**Cyanobacterium**	0.00	0.00	0.00	0.00	0.00	0.00	0.00	0.00	0.00	0.00	0.06	0.43	0.01	0.26	0.00	0.00	0.00	0.00	0.00	0.00	0.00	0.00	0.00	0.00
	**unclassified**	0.00	0.02	0.04	0.02	0.00	0.02	0.00	0.01	0.00	0.00	0.51	0.50	0.79	0.01	0.01	0.01	0.00	0.00	0.03	0.00	0.00	0.00	0.00	0.00
**Oscillatoriales**	**Coleofasciculus**	0.00	0.00	0.00	0.03	0.00	0.00	0.00	0.21	0.04	0.87	0.00	0.00	0.00	0.00	0.00	0.89	0.00	0.00	0.00	0.00	0.00	0.18	0.59	0.85
	**Kastovskya**	0.00	0.00	0.00	0.14	0.00	0.00	0.00	0.00	0.00	0.00	0.00	0.00	0.00	0.00	0.00	0.00	0.00	0.00	0.00	0.00	0.00	0.00	0.00	0.00
	**Planktothricoides**	0.00	0.00	0.00	0.00	0.00	0.00	0.00	0.00	0.00	0.05	0.00	0.00	0.00	0.01	0.00	0.02	0.00	0.00	0.00	0.00	0.00	0.00	0.00	0.00
	**Limnoraphis**	0.00	0.00	0.00	0.00	0.00	0.00	0.00	0.00	0.00	0.00	0.00	0.00	0.00	0.00	0.00	0.00	0.00	0.00	0.00	0.00	0.00	0.00	0.00	0.00
	**Lyngbya**	0.00	0.00	0.00	0.62	0.93	0.00	0.00	0.00	0.00	0.00	0.00	0.00	0.00	0.00	0.00	0.00	0.83	0.00	0.00	0.00	0.00	0.01	0.00	0.00
	**Hydrocoleum**	0.00	0.00	0.01	0.12	0.00	0.00	0.00	0.03	0.03	0.00	0.00	0.00	0.00	0.52	0.00	0.00	0.03	0.00	0.00	0.00	0.00	0.00	0.00	0.00
	**Oscillatoria**	0.00	0.00	0.02	0.00	0.00	0.00	0.00	0.00	0.00	0.00	0.00	0.00	0.00	0.00	0.13	0.00	0.00	0.00	0.00	0.00	0.00	0.00	0.00	0.00
	**Pseudoscillatoria**	0.00	0.00	0.00	0.00	0.00	0.00	0.00	0.00	0.00	0.00	0.00	0.00	0.00	0.00	0.00	0.00	0.00	0.00	0.00	0.00	0.00	0.00	0.00	0.00
	**unclassified**	0.00	0.00	0.01	0.05	0.03	0.03	0.00	0.70	0.64	0.02	0.01	0.01	0.00	0.11	0.01	0.05	0.04	0.00	0.01	0.00	0.34	0.08	0.02	0.12
**Chroococcidiopsidales**	**unclassified**	0.00	0.00	0.00	0.00	0.00	0.00	0.00	0.00	0.00	0.00	0.00	0.00	0.00	0.00	0.00	0.00	0.00	0.00	0.00	0.00	0.00	0.00	0.00	0.00
**Nostocales**	**Nodularia**	0.00	0.00	0.07	0.00	0.00	0.00	0.00	0.00	0.00	0.00	0.00	0.00	0.00	0.00	0.00	0.00	0.00	0.00	0.00	0.00	0.00	0.00	0.00	0.00
	**Calochaete**	0.00	0.01	0.00	0.00	0.00	0.00	0.00	0.00	0.00	0.00	0.00	0.00	0.00	0.00	0.00	0.00	0.00	0.00	0.00	0.00	0.00	0.00	0.00	0.00
	**Anabaena**	0.86	0.15	0.00	0.00	0.00	0.00	0.00	0.00	0.00	0.00	0.00	0.00	0.00	0.00	0.00	0.00	0.00	0.00	0.00	0.00	0.00	0.00	0.00	0.00
	**Nostoc**	0.00	0.80	0.00	0.00	0.00	0.00	0.00	0.00	0.00	0.00	0.00	0.00	0.00	0.00	0.00	0.00	0.00	0.00	0.00	0.00	0.00	0.00	0.00	0.00
	**Hassallia**	0.00	0.00	0.01	0.00	0.00	0.00	0.00	0.00	0.00	0.00	0.00	0.00	0.00	0.00	0.00	0.00	0.00	0.00	0.00	0.00	0.00	0.00	0.00	0.00
	**unclassified**	0.04	0.01	0.05	0.00	0.00	0.00	0.01	0.00	0.00	0.00	0.00	0.00	0.00	0.00	0.00	0.00	0.00	0.01	0.00	0.00	0.01	0.00	0.00	0.00
**Pleurocapsales**	**Stanieria**	0.00	0.00	0.00	0.00	0.00	0.00	0.00	0.00	0.00	0.00	0.00	0.00	0.04	0.00	0.00	0.00	0.00	0.00	0.00	0.00	0.00	0.00	0.00	0.00
	**Foliisarcina**	0.00	0.00	0.00	0.00	0.00	0.00	0.00	0.00	0.00	0.00	0.06	0.00	0.00	0.00	0.00	0.00	0.00	0.00	0.00	0.00	0.00	0.00	0.00	0.00
	**unclassified**	0.00	0.01	0.00	0.01	0.00	0.03	0.06	0.02	0.00	0.01	0.21	0.01	0.13	0.02	0.00	0.02	0.00	0.96	0.77	0.00	0.00	0.00	0.00	0.02
**Spirulinales**	**Spirulina**	0.00	0.00	0.00	0.00	0.00	0.00	0.00	0.00	0.27	0.00	0.00	0.00	0.00	0.00	0.61	0.00	0.00	0.00	0.00	0.00	0.00	0.00	0.00	0.00
	**unclassified**	0.00	0.00	0.00	0.00	0.00	0.00	0.00	0.00	0.01	0.00	0.05	0.00	0.00	0.01	0.04	0.00	0.00	0.00	0.00	0.00	0.00	0.00	0.00	0.00
**Synechococcales**	**Prochlorococcus**	0.00	0.00	0.00	0.00	0.00	0.04	0.00	0.00	0.00	0.00	0.00	0.00	0.00	0.00	0.01	0.00	0.00	0.00	0.12	0.00	0.00	0.00	0.00	0.00
	**Cyanobium**	0.08	0.00	0.00	0.00	0.00	0.23	0.00	0.00	0.00	0.00	0.00	0.00	0.00	0.00	0.01	0.00	0.00	0.00	0.00	0.00	0.00	0.00	0.00	0.00
	**Dactylococcopsis**	0.00	0.00	0.00	0.00	0.00	0.00	0.00	0.00	0.00	0.00	0.00	0.00	0.00	0.00	0.00	0.00	0.00	0.00	0.00	0.89	0.49	0.40	0.34	0.00
	**unclassified**	0.01	0.00	0.76	0.02	0.03	0.66	0.92	0.02	0.01	0.03	0.05	0.00	0.00	0.04	0.19	0.00	0.09	0.01	0.06	0.10	0.14	0.32	0.04	0.02
**unclassified Cyanobacteria**		0.00	0.00	0.00	0.00	0.00	0.00	0.00	0.00	0.00	0.00	0.01	0.02	0.02	0.03	0.00	0.00	0.00	0.02	0.00	0.00	0.00	0.00	0.00	0.00

Iceland (IC), Germany (DE), France (FR), Croatia (CR), Oman (OM). Color gradient indicate high abundance (red) to low abundance (white), and absence (grey) of taxa within each sample.
