# Supplementary figures and images for: Latitudinal gradient of cyanobacterial diversity in tidal flats
Source: PLoS One. 2019 Nov 13;14(11):e0224444. doi: 10.1371/journal.pone.0224444 (PMC6853291; doi:10.1371/journal.pone.0224444)

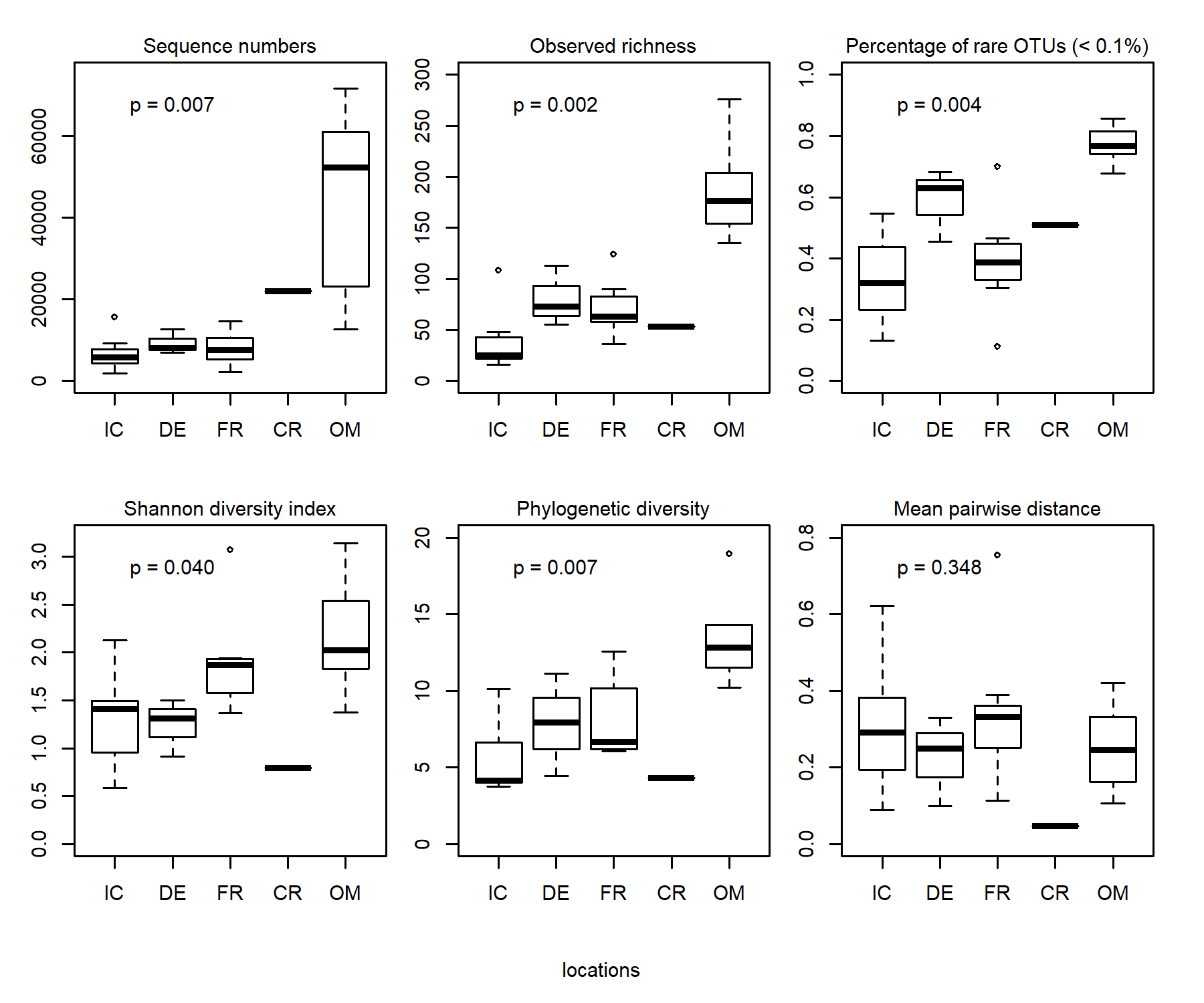

Supplement: S1 Fig — Boxplots of sequence numbers, alpha diversity estimated and percentage of rare OTUs per location. OTU-based diversity is described as observed richness and Shannon diversity index, phylogeny-based diversity is described as Faith’s phylogenetic diversity (PD) and the abundance weighted mean pairwise distance (MPD) based on the Maximum likelihood tree of representative sequences per OTU; rare OTUs contain less than 0.1% of all sequences per sample; CR = Croatia, FR = France, DE = Germany, IC = Iceland, OM = Oman, p-values of Kruskal-Wallis tests were shown. (TIF) [file pone.0224444.s008.tif]

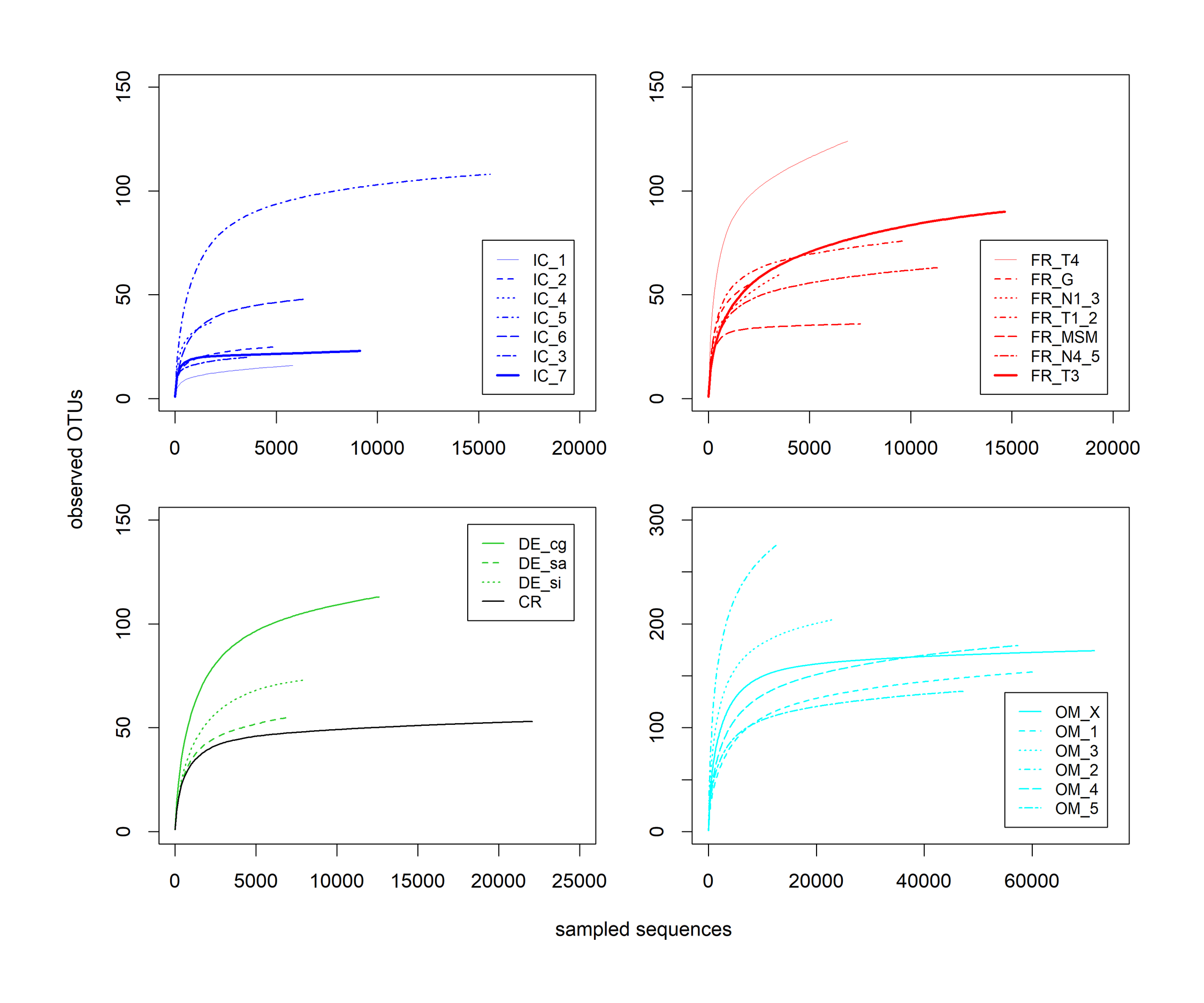

Supplement: S2 Fig — Rarefaction curves were calculated with 1000 randomizations based on all quality checked sequences per sample using a re-sampling without replacement approach. Colors code locations: dark blue = Iceland (IC), red = France (FR), black = Croatia (CR), green = Germany (DE), light blue = Oman (OM). (TIF) [file pone.0224444.s009.tif]

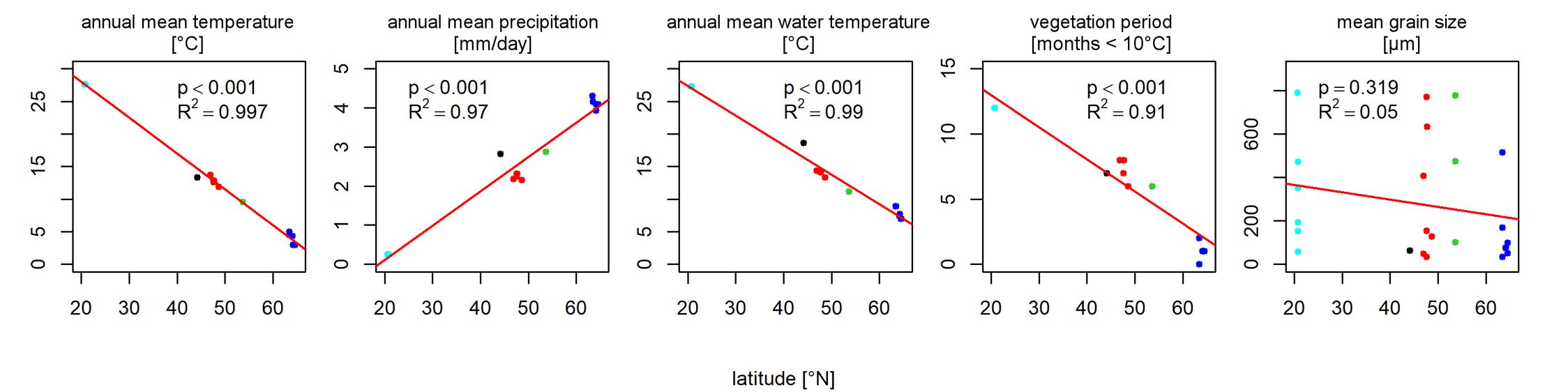

Supplement: S3 Fig — Colors code locations: dark blue = Iceland (IC), red = France (FR), black = Croatia (CR), green = Germany (DE), light blue = Oman (OM). (TIF) [file pone.0224444.s010.tif]

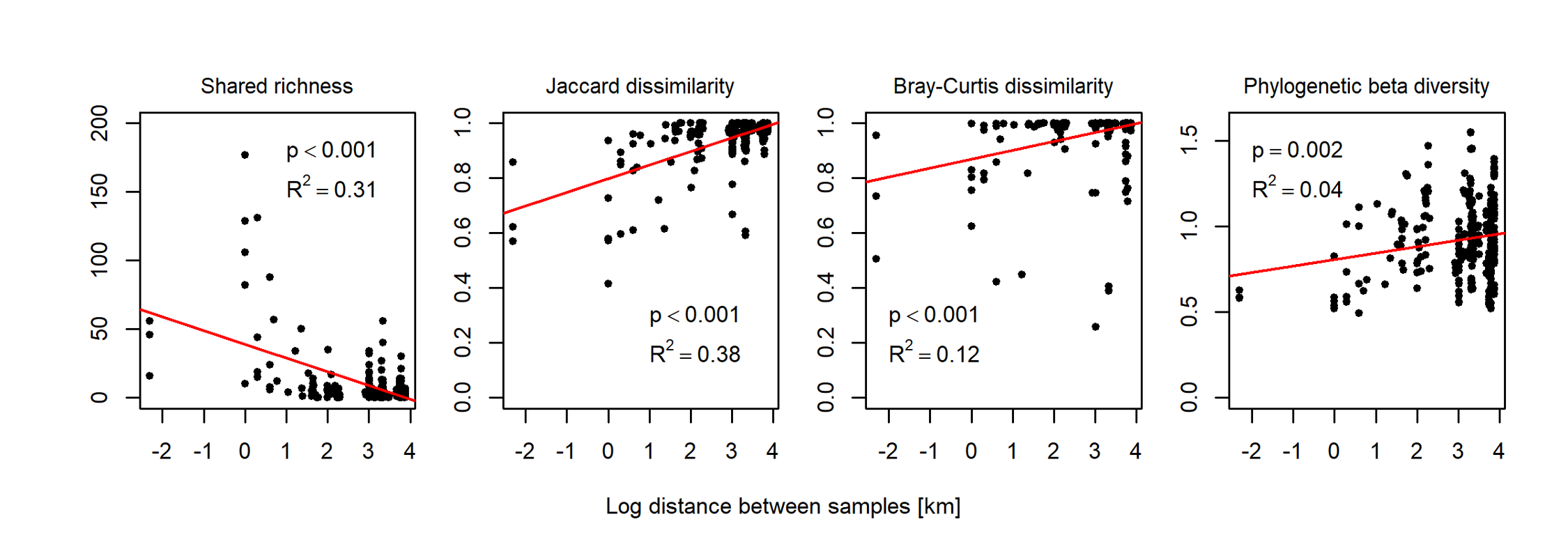

Supplement: S4 Fig — Beta diversity is described as shared richness, Jaccard and Bray-Curtis dissimilarity coefficients (OTU-based), and phylogenetic beta diversity (MPD), distance was previously log transformed. (TIF) [file pone.0224444.s011.tif]

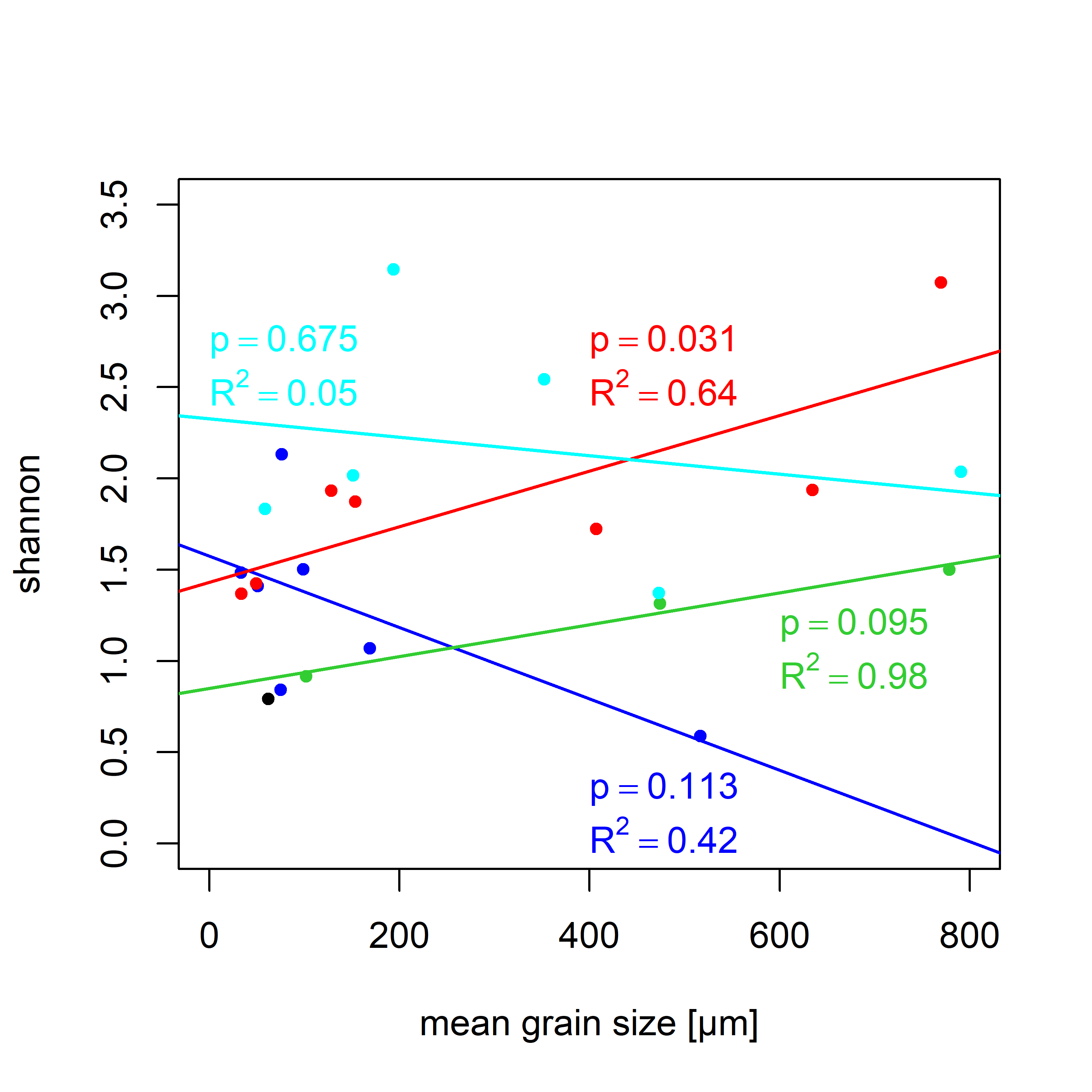

Supplement: S5 Fig — Colors code locations: dark blue = Iceland (IC), red = France (FR), black = Croatia (CR), green = Germany (DE), light blue = Oman (OM). (TIF) [file pone.0224444.s012.tif]

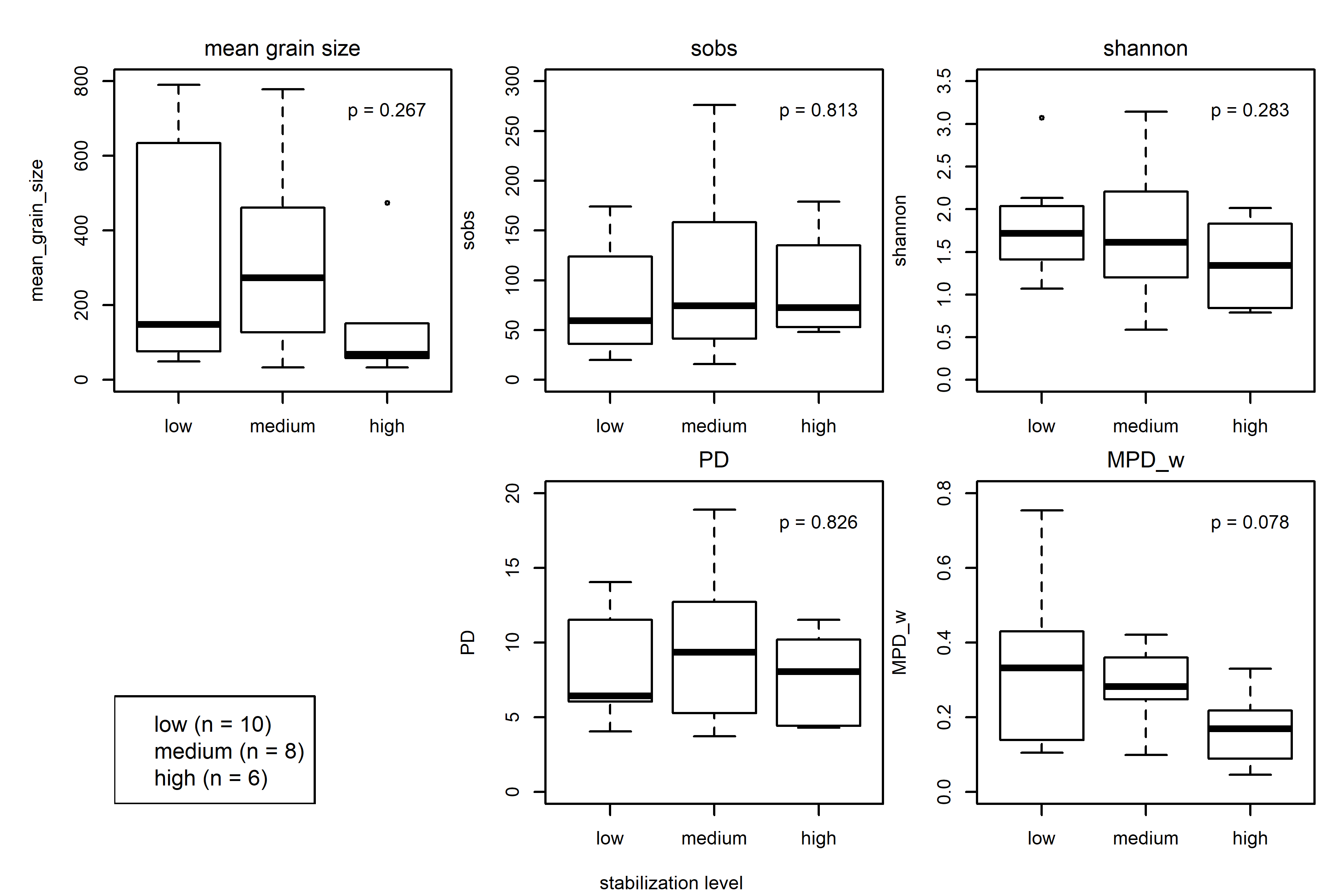

Supplement: S6 Fig — Diversity was described as observed richness (sobs), Shannon diversity index (shannon), Faith’s phylogenetic diversity (PD), and the abundance weighted mean pairwise distance (MPD) based on the Maximum likelihood tree of representative sequences. The stabilization level of all samples was qualitatively classified into three classes: high (well-stabilized and laminated microbial mats, that can be peeled off the sediment in large pieces (> 25 cm2)), medium (slightly stabilized sediment without clear laminations, that cannot be peeled off the sediment in large, but smaller pieces), and low (lose sediment without notably amounts of stabilizing compounds). p-values of Kruskal-Wallis tests were shown. (TIF) [file pone.0224444.s013.tif]
